# Improving cell mixture deconvolution by identifying optimal DNA methylation libraries (IDOL)

**DOI:** 10.1186/s12859-016-0943-7

**Published:** 2016-03-08

**Authors:** Devin C. Koestler, Meaghan J. Jones, Joseph Usset, Brock C. Christensen, Rondi A. Butler, Michael S. Kobor, John K. Wiencke, Karl T. Kelsey

**Affiliations:** Department of Biostatistics, University of Kansas Medical Center, 3901 Rainbow Blvd., Kansas City, 66160 KS USA; Centre for Molecular Medicine and Therapeutics, Child and Family Research Institute, Department of Medical Genetics, The University of British Columbia, 950 West 28th Ave., Vancouver, V5Z 4H4 BC Canada; Department of Epidemiology, Geisel School of Medicine, Dartmouth College, 1 Medical Center Dr., Lebanon, 03756 NH USA; Department of Pharmacology and Toxicology, Dartmouth College, 1 Rope Ferry Rd., Hanover, 03755 NH USA; Department of Community and Family Medicine, Geisel School of Medicine, Dartmouth College, 1 Medical Center Dr., Lebanon, 03756 NH USA; Department of Pathology and Laboratory Medicine, Brown University, 70 Ship St., Providence, 02912 RI USA; Department of Neurological Surgery, University of California San Francisco, 505 Parnassus Ave., San Francisco, 94143 CA USA; Department of Epidemiology, Brown University, 121 South Main St., Providence, 02912 RI USA

**Keywords:** EWAS, DNA methylation, Cell mixture estimation, Cell heterogeneity

## Abstract

**Background:**

Confounding due to cellular heterogeneity represents one of the foremost challenges currently facing Epigenome-Wide Association Studies (EWAS). Statistical methods leveraging the tissue-specificity of DNA methylation for deconvoluting the cellular mixture of heterogenous biospecimens offer a promising solution, however the performance of such methods depends entirely on the library of methylation markers being used for deconvolution. Here, we introduce a novel algorithm for ***Id***entifying ***O***ptimal ***L***ibraries (***IDOL***) that dynamically scans a candidate set of cell-specific methylation markers to find libraries that optimize the accuracy of cell fraction estimates obtained from cell mixture deconvolution.

**Results:**

Application of IDOL to training set consisting of samples with both whole-blood DNA methylation data (Illumina HumanMethylation450 BeadArray (HM450)) and flow cytometry measurements of cell composition revealed an optimized library comprised of 300 CpG sites. When compared existing libraries, the library identified by IDOL demonstrated significantly better overall discrimination of the entire immune cell landscape (*p* = 0.038), and resulted in improved discrimination of 14 out of the 15 pairs of leukocyte subtypes. Estimates of cell composition across the samples in the training set using the IDOL library were highly correlated with their respective flow cytometry measurements, with all cell-specific *R*^2^>0.99 and root mean square errors (*RMSE*s) ranging from [0.97 % to 1.33 %] across leukocyte subtypes. Independent validation of the optimized IDOL library using two additional HM450 data sets showed similarly strong prediction performance, with all cell-specific *R*^2^>0.90 and *RMSE*<4.00 *%*. In simulation studies, adjustments for cell composition using the IDOL library resulted in uniformly lower false positive rates compared to competing libraries, while also demonstrating an improved capacity to explain epigenome-wide variation in DNA methylation within two large publicly available HM450 data sets.

**Conclusions:**

Despite consisting of half as many CpGs compared to existing libraries for whole blood mixture deconvolution, the optimized IDOL library identified herein resulted in outstanding prediction performance across all considered data sets and demonstrated potential to improve the operating characteristics of EWAS involving adjustments for cell distribution. In addition to providing the EWAS community with an optimized library for whole blood mixture deconvolution, our work establishes a systematic and generalizable framework for the assembly of libraries that improve the accuracy of cell mixture deconvolution.

**Electronic supplementary material:**

The online version of this article (doi:10.1186/s12859-016-0943-7) contains supplementary material, which is available to authorized users.

## Background

The past decade has witnessed an exponential increase in epidemiologic studies of DNA methylation, driven in large part by increasing appreciation for its critical role in the development and progression of human diseases together with the declining cost of high-throughput technologies for interrogating the epigenome. Following the namesake adopted for genome-wide, genetic association studies of disease phenotypes (GWAS), studies investigating the role of DNA methylation in human diseases and exposures have been aptly dubbed epigenome-wide association studies (EWAS) [1]. While GWAS and EWAS data share many of the same analytical challenges, the tissue specificity of DNA methylation presents an added layer of complexity in the analysis, and particularly in the interpretation of EWAS. Owing to the tissue specificity of DNA methylation, it is now well established that comparisons of methylation signatures assessed over heterogenous cell populations are susceptible to confounding and misinterpreted associations [2–5], issues that are believed by many to be among the foremost challenges currently facing EWAS [6–9].

Recent attempts aimed at minimizing the potential for confounding in the analysis of DNA methylation data have prompted some researchers to restrict methylation assessment to purified cell populations [10, 11], for example, CD4+ or CD14+ cells isolated from peripheral blood. Although such studies may be less prone to confounding by leukocyte-lineage heterogeneity compared to those involving whole blood (WB) DNA methylation assessments, purification of cell populations carrying these markers will not completely eliminate heterogeneity attributable to lineage differences [3]. Other attempts to address the potential for confounding in blood-based DNA methylation data have involved adjusting statistical models with additional terms reflecting the cell composition of study samples using, for example, measurements from complete blood cell counts (CBC) or fluorescence-activated cell sorting (FACS) [5, 12]. However, these measurements are not often collected as part of EWAS (Additional file 1: Table S1), the reasons for which commonly include: insufficient quantities of substrate for both DNA methylation assessment and measurements of cell composition, budgetary constraints, and the inability of technologies - such as FACS - to accurately measure biospecimens stored over extended time periods. In addition, because EWAS typically represent subsidiary studies, whose associated parent study predate current understanding of the impact of cellular heterogeneity on DNA methylation analyses, direct measurements of cell composition were unlikely to have been performed when biospecimens were initially collected. These considerations, together with the emerging consensus concerning the need to account for cell composition in the statistical analysis of DNA methylation data [6–9] have served to motivate the development of novel statistical/bioinformatic methodologies for addressing the potential confounding effects driven by cell heterogeneity [13–15]. The first of such methodologies [13] and the most widely applied within the EWAS literature leverages the cell-specificity of DNA methylation as the basis for estimating the cellular landscape of samples consisting of heterogeneous cell populations. This approach, commonly referred to as cell mixture deconvolution (CMD), is grounded on the assumption that the methylation signature for a given target sample (methylation profiled across a diverse population of underlying cell types) can be viewed as a weighted mixture of the unique methylation signature of each of its constituent cell types, with weights reflecting the proportion of each cell type within the target biospecimen. Under certain constraints, fairly routine statistical procedures can be employed to estimate such weights, thereby providing investigators with a “prediction” of the cellular distribution for each target sample to which it is applied. Much in the same way one would adjust for cell composition if cell fractions were measured directly, estimates of cell composition obtained using CMD can be added as additional covariate terms to control for the potential confounding effects associated with cell heterogeneity [16–21].

The first and most critical step of CMD and the impetus for this research, involves assembling a library of cell-specific methylation biomarkers that collectively reflect the unique methylomic fingerprint of each cell type. In the case of leukocyte subtypes, we refer to such cell-specific methylation biomarkers as leukocyte-differentially methylated regions (L-DMRs) to convey their differential methylation status across leukocyte subtypes. Motivated by the critical role played by L-DMR libraries and their relationship to the accuracy of cell composition estimates [8, 22], here we develop and evaluate a novel, iterative algorithm for Identifying Optimal L-DMR Libraries (IDOL) that improves the accuracy and efficiency of cell composition estimates obtained by CMD.

In what follows, we aim to address three key questions: (*i*) does the optimal library identified from IDOL result in improved estimates of cellular composition compared to existing libraries, (*ii*) if so, are there discernible differences between libraries that might offer an explanation for their prediction performance, and lastly (*iii*), what impact does the difference in prediction performance between libraries have on EWAS when estimates of cell mixture are desired. To address these important questions we begin by applying IDOL to a training set consisting of samples with both whole-blood DNA methylation data (assayed using the Illumina HumanMethylation450 BeadArray (HM450)) and flow cytometry measurements of cell composition in order to calibrate the selection of an optimal L-DMR library. To illustrate the utility of the identified IDOL library as resource for future EWAS, we benchmark its performance against existing libraries in two independent HM450 data sets and conduct a thorough comparison of libraries to gain insight into their observed prediction accuracy. Finally, the impact of different libraries on the false positive rate and statistical power of EWAS is evaluated using both simulation studies and a data application involving two large publicly available HM450 data sets.

## Results

**Fig. 1:**
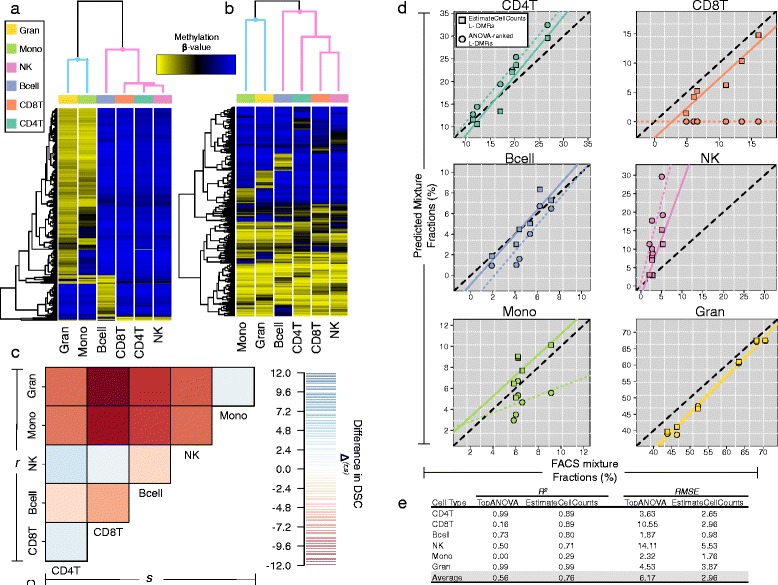
Impact of L-DMR library on the accuracy of cell composition estimation. **a**, **b** Hierarchical clustering heat maps of the mean methylation signatures of isolated leukocyte subtypes [3] using (**a**) the top 600 ANOVA-ranked L-DMRs (TopANOVA library) and (**b**) the 600 L-DMRs that uniquely distinguish each cell type from all other cell types (EstimateCellCounts default library). Column dendrograms are colored to reflect the cell-lineage of leukocyte subtypes: lymphocytes (*pink*) and myeloid-derived cells (*blue*). **c** Image plot showing the difference in the dispersion separability criterion (DSC) between the EstimateCellCounts and TopANOVA libraries. For a given pair of leukocyte subtypes, larger values of DSC difference (shades of blue) indicate better discrimination associated with the EstimateCellCounts library, whereas smaller values of DSC difference (shades of red) indicate better discrimination associated with the TopANOVA library. **d** Scatterplots of the CMD predicted and FACS cell fractions for the *n*=6 AdultMixed samples. Dashed lines indicate the line of unity, dotted lines represent the fitted regression lines based on cell predictions obtained using the TopANOVA library, and solid lines represent the fitted regression lines based on cell predictions obtained using the EstimateCellCounts library. **e** Cell-specific prediction performance for the AdultMixed samples based on the TopANOVA and EstimateCellCounts libraries

The essential nature of library assembly and its impact on the accuracy of cell composition estimates is highlighted in Fig. 1. Figure 1a,b depict heat maps generated from hierarchical clustering the *K*=6 major leukocyte components of WB (i.e., CD4T cells, CD8T cells, natural killer (NK) cells, B cells, monocytes, and granulocytes) based on their methylation signature across two different L-DMR libraries [13, 23]. The first of these libraries to appear in the literature (TopANOVA [13]) was assembled using the 600 CpGs with the largest *F*-statistics computed from a series of ANOVA models comparing CpG-specific patterns of methylation across leukocytes (Fig. 1a). The second library is the default library used by the EstimateCellCounts function in the *minfi* Biocondutor package [23]. While also comprised of 600 CpGs, the EstimateCellCounts library is instead assembled using the top 100 CpGs that uniquely distinguish each cell type from the remaining *K*−1 cell types (100×*K*=600 Total CpGs). While both libraries adequately discriminate lymphoid-derived cells (CD4T, CD8T, NK, and B cells) from myeloid-derived cells (monocytes and granulocytes), the EstimateCellCounts library exhibits far better discrimination of lineage-specific cell types, particularly, NK, CD4T, and CD8T lymphocytes (Fig. 1c). The net result of its improved discrimination of lineage-specific subtypes is uniformly better prediction performance across the entire immune cell landscape, the largest of such gains being associated with NK, CD4T, and CD8T lymphocytes (Fig. 1d,e, Additional file 2: Figure S1).

The principle reason for the difference in discrimination power and prediction accuracy between libraries is due entirely to the criteria used for their assembly. While assembling libraries using ANOVA *F*-statistics might seem reasonable, it is inherently susceptible to the over-selection of CpGs that are capable of discriminating certain subsets of leukocytes (i.e., lymphocytes versus myeloid cell types), but provide poor discrimination of other subsets (i.e., lineage-specific subtypes). On the other hand, the EstimateCellCounts library is constructed by imposing an equal representation of CpGs for each cell type (top 100 cell-specific L-DMRs). This strategy leads to better discrimination of lineage-specific cell types and, as a result, improved estimation accuracy of those cell types (Fig. 1d,e). Despite representing an obvious improvement over TopANOVA, the prediction accuracy associated with EstimateCellCounts demonstrates ample room for improvement and suggests that further refinements in the assembly of L-DMR libraries may provide the solution.

**Fig. 2:**
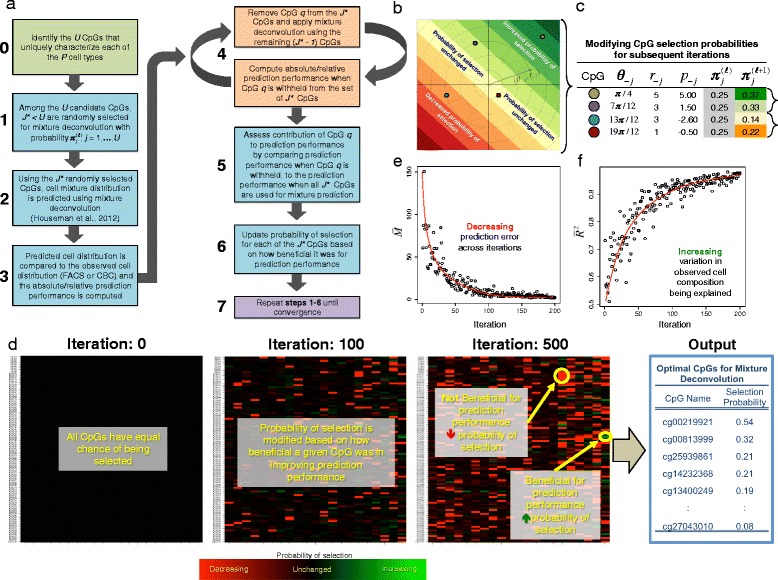
Conceptual illustration of the IDOL algorithm. **a** Schematic diagram showing each step of IDOL. **b**, **c** Illustration of the scheme for updating the selection probabilities of L-DMRs. **d** Conceptual depiction of the L-DMR selection probabilities as a function of the sequential progression of IDOL. At iteration 0, L-DMRs have an equal probability of being selected for inclusion in the randomly assembled L-DMR subset. At each sequential iteration of IDOL (i.e., moving from left to right), the selection probabilities for L-DMRs are updated in a manner proportion to their contribution to prediction performance; selection probabilities for L-DMRs that contribute favorably to prediction performance are increased (increasing shades of green), whereas the selection probabilities for those that hinder prediction performance are decreased (increasing shades of red). Upon algorithm termination, the *J* ^⋆^ L-DMRs with the largest selection probabilities are taken to represent the optimal L-DMR library. **e**, **f** Plots showing mean *RMSE* ($\bar {M}$) and coefficient of determination ($\bar {R}^{2}$) respectively, as a function of sequential progression of the the IDOL algorithm

Motivated by the critical role played by L-DMR libraries on the accuracy of cell composition estimates, we focus here on the development and evaluation of a novel iterative algorithm (IDOL) for identifying L-DMR libraries that improve the performance of CMD. A schematic diagram illustrating the various steps of IDOL is given in Fig. 2a. IDOL first involves the construction of a candidate set of L-DMRs consisting of CpG sites exhibiting differential DNA methylation across leukocyte subtypes. From this candidate set, subsets of L-DMRs are randomly selected at each iteration, with each randomly selected L-DMR being evaluated for its contribution to cell composition prediction accuracy. The contribution of each L-DMR is then used to modify its probability of selection in subsequent rounds of IDOL, where selection probabilities are updated in a manner proportional to its contribution to prediction accuracy. This is similar in principle to the weight updating rule in supervised competitive learning networks and the update rules employed in Learning Vector Quantization [24]. Specifically, L-DMRs found to contribute favorably to prediction performance are updated to have greater chance of being selected in subsequent iterations, whereas L-DMRs that hinder or have no effect on prediction performance are updated to have a reduced chance of being selected (Fig. 2b,c). This dynamic process is repeated thousands of times, with L-DMR selection probabilities evolving at each iteration depending on how they impact the accuracy of CMD estimates (Fig. 2d). By updating selection probabilities in this way, randomly selected L-DMR subsets at each sequential IDOL iteration become enriched with L-DMRs that were previously marked as beneficial to prediction accuracy. As a result, the temporal evolution of IDOL witnesses the preferential selection of L-DMR subsets that, as a whole, contribute to improved accuracy of CMD estimates (Fig. 2e,f). Upon termination, one is left with the subset consisting of the L-DMRs with the largest selection probabilities, henceforth referred to as the optimal IDOL library.

### Training the selection of L-DMR libraries for cell mixture deconvolution

**Fig. 3:**
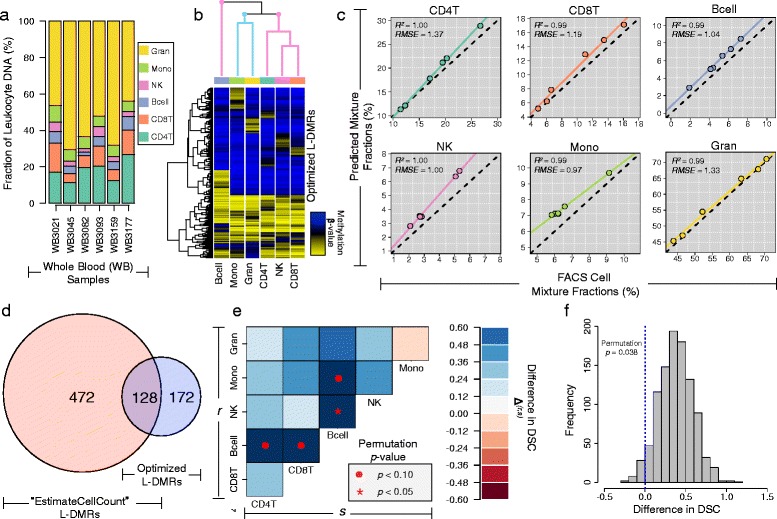
Results obtained from applying IDOL to the training set. **a** Stacked bar plots showing the FACS measured fractions of granulocytes (Gran), monocytes (Mono), natural-killer cell (NK), B cells (Bcell), CD8T lymphocytes (CD8T), and CD4T lymphocytes (CD4T) across the 6 training samples. **b** Hierarchical clustering heat map of the mean methylation signature of leukocyte cell-types (columns) based on the 300 optimized L-DMRs (rows) identified by IDOL. The column dendrogram is colored to reflect the cell lineage of the leukocyte subtypes, where lymphocyte-derived subtypes are colored pink and myeloid-derived cell types are colored blue. **c** Scatterplots of FACS measured cell fractions (x-axes) and predicted cell proportions obtained using the optimized IDOL library (y-axes). Dotted lines indicate the line of unity and colored lines represent the regression line fit to the FACS measured cell fractions and predicted cell fractions. **d** Overlap between IDOL and EstimateCellCounts libraries. **e** Image plot showing the difference in the dispersion separability criterion (DSC) between the IDOL and EstimateCellCounts libraries for discriminating specific pairs of leukocyte subtypes. For a given pair of leukocytes, larger values of DSC difference (shades of blue) indicate better discrimination associated with the IDOL library, whereas smaller values of DSC difference (shades of red) indicate better discrimination associated with the EstimateCellCounts library. **f** Histogram showing the results of a permutation-based testing procedure for examining the difference in the overall DSC between the IDOL and EstimateCellCounts libraries

To calibrate the selection of optimal L-DMR libraries, we first applied IDOL to a training data set consisting *n*=6 non-diseased adults with WB DNA methylation and immune profiling data (Section ‘Adult whole blood (WB) samples’). Flow cytometry estimated cell fractions of CD4T, CD8T, natural killer (NK), B cells, monocytes, and granulocytes across the training samples are depicted in Fig. 3a. On average, granulocytes represented the most abundant cell type across the training samples (mean =57.4 *%*, sd =11.5 *%*), followed by CD4T (mean =17.9 *%*, sd =5.7 *%*), CD8T (mean =9.7 *%*, sd =4.5 *%*), monocytes (mean =6.7 *%*, sd =1.2 *%*), B cells (mean =4.9 *%*, sd =1.9 *%*), and NK cells (mean =3.5 *%*, sd =1.4 *%*), in descending order of abundance.

Since the objective of IDOL is to identify the best subset (or subsets) of L-DMRs from a larger candidate set of putative L-DMRs, we first focused on constructing this candidate set by identifying CpG sites with differential methylation across leukocytes. Using the DNA methylation profiles for isolated leukocyte subtypes reported in [3], we first fit a series of two-sample *t*-tests to compare CpG-specific DNA methylation patterns across the *K*=6 immune cell subtypes. Specifically, the CpG-specific methylation signature of each of cell type was compared to the *K*−1 remaining cell types and the top 150 CpGs with largest and smallest *t*-statistics were combined into a single candidate list consisting of 1,800 putative L-DMRs (Additional file 3: Table S2). Following construction of the candidate set, we next applied IDOL to identify optimal libraries across a range of possible library sizes, from 100 to 800 CpG loci in increments of one-hundred. Across the spectrum of library sizes considered, the average *R*^2^ and root mean square error (*RMSE*) between flow cytometry measurements and predicted cell type proportions obtained from the identified optimal libraries was very stable, ranging from 0.98 to 1.00 for *R*^2^ and from 2.41 % to 3.30 % for *RMSE* (Additional file 4: Figure S2). As noted in Additional file 4: Figure S2, a subtle drop-off in prediction performance was observed libraries whose size exceeded 500 CpGs. Given the general preference for prediction models that use fewer features and because the library consisting of 300 CpGs (Additional file 5: Table S3) performed favorably both with respect to its average *R*^2^ and *RMSE*, this library was selected as the representative IDOL library for all subsequent comparisons and analyses.

Hierarchical clustering of leukocytes based on their mean methylation signature across the 300 CpGs in the optimal IDOL library is given in Fig. 3b and clearly shows better discrimination of lymphocyte subtypes compared to the TopANOVA library (Fig. 1a). Using the IDOL library for deconvoluting the cellular mixture of the training set samples revealed a high degree of concordance between flow cytometry and predicted cell type proportions, with nearly perfect *R*^2^ values across all cell types and *RMSEs* ranging from as low as 0.97 % for monocytes to 1.37 % for CD4T cells (Fig. 3c). Across the six leukocytes, the average *R*^2^ and *RMSE* between the predicted and flow cytometry cell type proportions were estimated at 0.99 and 1.15 %, respectively. When compared to the results obtained from the application of both the EstimateCellCounts and TopANOVA libraries to training set (Fig. 1d,e, Additional file 2: Figure S1), the IDOL library resulted better prediction performance for all cell types except B cells, whose predictions from EstimateCellCounts exhibited slightly lower *RMSE* (0.98 % versus 1.04 %). Upon further comparison, the greatest improvements in prediction performance associated with the IDOL library occurred for monocytes and among lymphocyte subtypes. Specifically, the IDOL library resulted in monocyte predictions that explained approximately 70 % more variation in the flow cytometry measurements of monocytes compared to EstimateCellCounts (Figs. 1e and 3c). Similarly, predictions of CD4T, CD8T, and NK cell type fractions obtained from the IDOL library explained an average of 17 % more variation in the flow cytometry derived fractions of these cell types compared to EstimateCellCounts, and were associated with *RMSE*s that were on average 3.3-fold lower.

Of the 300 CpGs encompassing the IDOL library, 128 (43 %) were shared with 600 L-DMRs used by EstimateCellCounts (Fig. 3d, Additional file 5: Table S3). To understand how differences between these two libraries might explain their observed prediction performance, we next compared libraries with respect to their ability to discriminate specific pairs of leukocytes. For each library we computed the dispersion separability criterion (DSC), defined here as the ratio of the average distance between cell-specific centroids and the overall mean to the average distance between samples of the same cell type. As such, increasing DSC values indicate greater between-cell-type dispersion/discrimination. Using the leukocyte-specific methylation data reported in [3] as the basis for estimation, we found that the IDOL library resulted in a significantly larger DSC compared to the EstimateCellCounts library (permutation *p* =0.038) (Fig. 3f). Furthermore, a comparison of the DSC values computed between each pair of leukocytes showed that the IDOL library resulted in larger DSC values in 14 out of the 15 comparisons, of which 4 were associated with *p*-values that bordered on statistical significance (*p*<0.10) (Fig. 3e). Among the 4 comparisons with marginally statistically significant *p*-values, 3 involved specific pairs of lymphocyte subtypes.

### Independent validation of the optimal L-DMR set

**Fig. 4:**
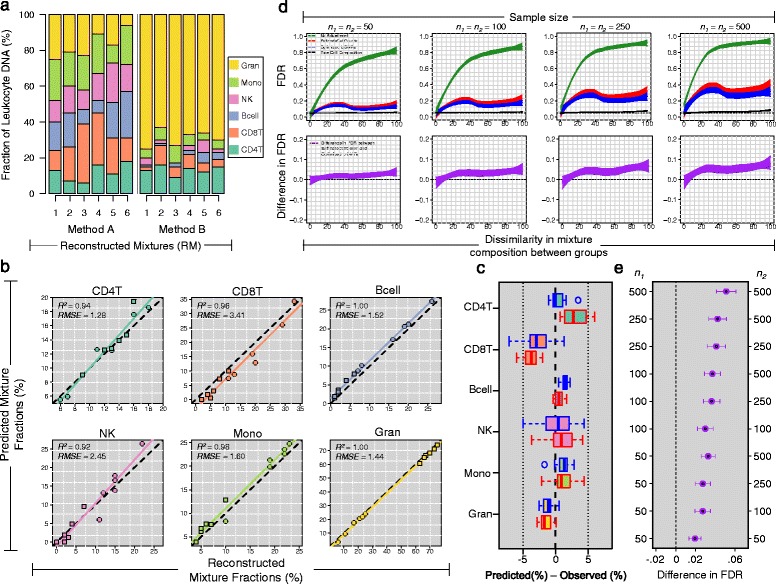
Results obtained from applying the optimal IDOL library to the testing sets. **a** Stacked bar plots showing the cell type fractions for each testing set sample. **b** Scatter plots of the true reconstructed mixture fractions (x-axes) and the predicted cell fractions obtained using the optimized IDOL library (y-axes). Circles indicate Method A samples and squares indicate Method B samples. Dotted lines indicate the line of unity and colored lines represent the regression line fit to the true reconstructed mixture fractions and predicted cell fractions. **c** Box plots showing the predicted cell (%) − observed cell (%) across leukocyte cell types, where blue boxes represent estimates obtained from the optimal IDOL library and red boxes represent estimates obtained from the EstimateCellCounts library. (**d**, top panel) Estimated false discovery rate (FDR) for a two-group comparison of DNA methylation as a function of the dissimilarity in the cellular distribution between groups (x-axes). Colored lines represent different approaches for cell composition adjustment. (**d**, bottom panel) Difference in the FDR between the EstimateCellCounts and IDOL libraries where points above the dotted line indicate that the EstimateCellCounts library resulted in more false positive results compared to the IDOL library. **e** Mean difference in the FDR for varying sample sizes when cell mixture was adjusted using cell fractions estimates from the EstimateCellCounts and IDOL libraries. Bars represent the 95 % bootstrap confidence intervals for each point estimate. Points to the right of the dotted line indicate that the EstimateCellCounts library resulted in more false positive results compared to the IDOL librarys

To validate the IDOL library identified in the training set, we next examined its performance for accurately deconvoluting the cellular composition of 12 additional samples spread across two independent test sets: MethodA and MethodB sets. As described in Section ‘Cell mixture reconstruction experiment’, the MethodA and MethodB data sets were created by mixing purified leukocyte subtype DNA from CD4T, CD8T, NK, B cells, monocytes, and granulocytes in predetermined proportions (Fig. 4a). As such, the true cellular mixture of the MethodA and MethodB samples are known with a high degree of confidence, representing ideal candidates in which to validate the optimal library identified by IDOL in its application to the training set.

As noted in Fig. 4a, whereas the MethodA samples are characterized by a roughly equivalent fraction of each cell type (mean CD4T =11.8 *%*; CD8T =20.8 *%*; NK = 15.0 %; Bcell =16.0 *%*, monocyte =19.2 *%*, and granulocyte =17.2 *%*), the cellular composition of the MethodB samples were reconstructed to resemble the immune cell landscape observed in healthy human adults [25] (mean CD4T =13.2 *%*; CD8T =6.0 *%*; NK =3.0 *%*; Bcell =2.7 *%*, monocyte =6.2 *%*, and granulocyte =69.0 *%*). Similar to the results obtained in the training set, cell type predictions in the testing sets using the IDOL library were highly correlated with true mixture fractions (Fig. 4b). Specifically, the cell-specific *R*^2^ values computed across both testing sets ranged from 0.94 (CD4T cells) to 1.00 (both, granulocytes and B cells), with an *R*^2^ of 0.97 averaged across the six cell types. In addition, the cell-specific *RMSEs* computed across the testing sets showed that in 4 out of the 6 cell types, predictions were, on average, within 2.0 % of their true reconstructed mixture proportions. The two exceptions being NK cells (*RMSE* =2.5 *%*) and CD8T cells (*RMSE* =3.4 *%*). A comparison of the cell-specific *R*^2^ and *RMSE*s computed within the MethodA and MethodB data sets separately revealed relatively minor differences in prediction accuracy (Additional file 6: Table S4 and Additional file 7: Figure S3 and Additional file 8: Figure S4). For the MethodA set, cell-specific *R*^2^ and *RMSE* ranged between [0.86, 1.00] and [1.09 %, 4.11 %] with mean values of 0.96 and 2.14 %, respectively. Similarly, in the MethodB data set, cell-specific *R*^2^ and *RMSE* ranged between [0.82, 0.98] and [1.44 %, 2.52 %] with mean values of 0.91 and 1.68 %. Furthermore, there appeared to be no association between the prediction performance of a given cell type and its true underlying fraction in the MethodA and MethodB reconstructed mixture samples (Additional file 7: Figure S3, Additional file 8: Figure S4 and Additional file 9: Figure S5).

The prediction performance obtained using the IDOL library compared favorably to the performance associated with EstimateCellCounts, the predictions of which explained, on average, 2 % less variation in the underlying reconstructed mixture fractions compared to the IDOL library (Additional file 6: Table S4 and Fig. 4c). The largest difference in performance was observed for CD4T cells, whose IDOL associated predictions explained an estimated 12 % more variation in the reconstructed mixture proportions of CD4T cells and were associated with a 2-fold lower *RMSE* compared to EstimateCellCounts (Additional file 6: Table S4 and Fig. 4c).

### Implications of cell composition adjustment methodology for EWAS

In the overwhelming majority of the studies using CMD, estimates of immune cell fractions are first obtained for each study sample, followed by their inclusion as additional covariate terms in statistical models to control for the potential confounding effects of cellular heterogeneity [26–28]. For this reason, metrics such as *R*^2^ and *RMSE*, while providing a useful starting point for comparing different L-DMR libraries, say little about how the prediction error associated with a given library relates to its impact on the power and false discovery rate (FDR) of EWAS. With this in mind, we conducted a series of analyses aimed at examining how adjustments for cellular mixture in the statistical modeling of DNA methylation data impact the ability to correctly identify true negatives (FDR) and true positives (power).

To understand the consequences of prediction error in cell fraction estimates for EWAS, we first conducted a simulation study comparing the FDR when different strategies for cell composition adjustment were employed, namely, when cell fraction estimates were obtained using the IDOL and EstimateCellCounts libraries. For our simulations, we assumed simplistic study design that, typical of many EWAS, focused on the identification of differentially methylated CpG sites between two groups, i.e., case/control comparison. As described in Section ‘Simulation study comparing false discovery rates (FDR) across different cell composition adjustment techniques’, for each sample, methylation beta-values were simulated for a total of 10,000 CpGs, assuming within-group sample sizes that ranged from small/moderate (i.e., *n*={50,100}) to moderate/large (i.e., *n*={250,500}). Most importantly, while the underlying cellular composition was permitted to vary across groups, each cell type was assumed to have an identical methylation signature between groups: no group effect. As such, tests of CpG-specific differential methylation between groups with adjustments for cellular composition should not be rejected and therefore represent the basis for our estimates of FDR.

As expected, the FDR was appropriately controlled at 5 % when adjustments for cell composition were carried out using the “true simulated” cell distribution (Fig. 4d, black lines). On the other hand, a clear inflation in the FDR was observed when tests for differential methylation were unadjusted for cellular composition, the degree of inflation depending heavily on the between-group dissimilarity in cellular distribution (Fig. 4d, green lines). While a subtle inflation in FDR was observed when cell type adjustments were carried out using cell fraction estimates obtained from the IDOL (blue lines) and EstimateCellCounts (red lines) libraries, the IDOL library tended to result in a reduced number of false positive results across the spectrum of simulation conditions (Fig. 4d). This observation is more clearly illustrated in Fig. 4e which depicts the average difference in FDR computed between EstimateCellCounts and the IDOL libraries across the range of assumed within group sample sizes. Compared to EstimateCellCounts, the IDOL library resulted in, on average, 2 %–5 % fewer false discoveries when within-group sample sizes ranged from 50 to 500.

**Fig. 5:**
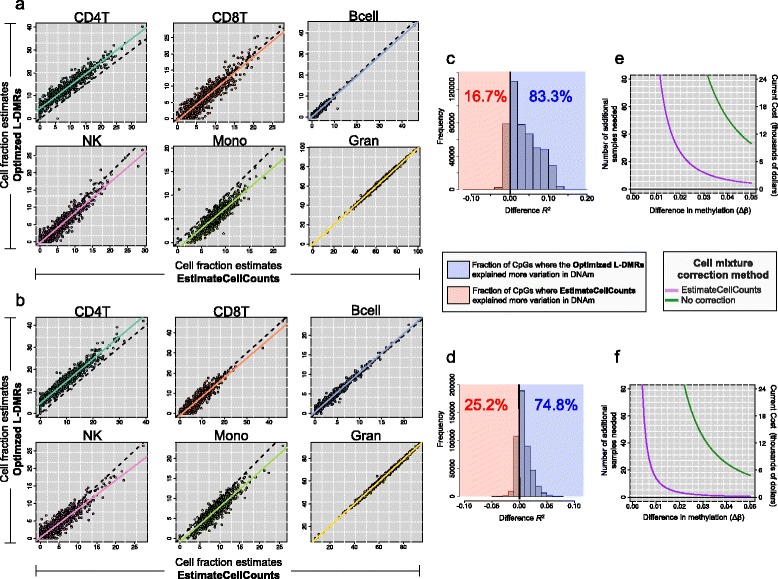
Cell mixture deconvolution of the Liu and Hannum blood data sets using the IDOL and EstimateCellCounts libraries. **a**, **b** Scatter plots of the predicted cell type fractions obtained using EstimateCellCounts library (x-axes) and the IDOL library (y-axes) for the Liu and the Hannum data sets, respectively. **c**, **d** Distribution of the difference in the *R* ^2^ computed from the IDOL and EstimateCellCounts libraries for the (**c**) Liu and (**d**) Hannum data sets. **e**, **f** Estimated number of additional samples needed (y-axis, left) and approximate additional cost (y-axis, right) as a function of the desired difference in DNA methylation to be detected (x-axis) when correction for cell mixture was carried out using the EstimateCellCounts library. Variance estimates were obtained from the (**e**) Liu and (**d**) Hannum data sets

To further understand the implications of cell type prediction methodology for EWAS, we made use of two of the largest publicly-available WB DNA methylation data sets [16, 29]. Our analysis of the Liu [16] and Hannum [29] data sets was aimed at addressing two different but related questions: (*i*) which cell prediction methodology performed better at explaining variation in DNA methylation within each data set and (*ii*) how does the additional variation being explained relate to the statistical power of each study. To address these questions we began by estimating the cellular distribution of the samples in each data set using both the IDOL and EstimateCellCounts libraries (Fig. 5a,b).

As noted in Fig. 5a,b, a high degree of correlation was observed in the cell fraction estimates obtained using the IDOL and EstimateCellCounts libraries, with cell-specific *R*^2^ ranging from [0.80, 0.99] and [0.84, 0.99] for the Liu and Hannum data sets, respectively. In both data sets, the predicted fraction of monocytes exhibited the greatest variation between the the IDOL and EstimateCellCounts libraries, with the IDOL library resulting in slightly smaller estimates of monocyte fractions compared to EstimateCellCounts, on average: (5.4 % versus 7.8 %) and (6.8 % versus 8.7 %) in the Liu and Hannum data sets, respectively. Conversely, estimates of CD4T cells obtained from the IDOL library were, on average, slightly larger compared to those obtained from EstimateCellCounts; (12.9 % versus 8.3 %) and (13.8 % versus 9.1 %) in the Liu and Hannum data sets.

Array-wide comparisons of the proportion of CpG-specific variation in DNA methylation explained by cell composition estimates revealed that the IDOL library tended to explain more variation compared to EstimateCellCounts (Fig. 5c,d). Specifically, cell estimates obtained from the IDOL library explained more variation in DNA methylation for 83.3 % of the CpGs in the Liu data set and 74.8 % of the CpGs in the Hannum data set, both of which represent significantly larger proportions than would be expected by random chance (permutation *p*<0.001 for both). To understand how these findings relate to statistical power of EWAS, we used the residual variance estimates obtained from each methodology as the basis for estimating the sample size required for detecting a statistically significant difference in DNA methylation at 80 % power (Section ‘Data application for exploring the implications of cell composition adjustment in EWAS’). Figure 5e,f show the number of additional samples needed when cell type correction was carried out using estimates from EstimateCellCounts (purple) or no cell type correction (green), as a function of the desired difference to be detected in the mean methylation beta-values between two groups. Using the residual variance estimates computed in the Hannum data set, there were only modest differences in the number of additional samples needed when cell type correction was based on estimates from EstimateCellCounts, with virtually no difference between the IDOL library and EstimateCellCounts beyond effect sizes of 0.03 (on the beta-value scale). However, using the residual variance estimates obtained in the Liu data set showed that, for effect sizes ranging from 0.03–0.05 (on the beta-value scale), approximately 15 and 5 additional samples respectively would be needed if the analysis was adjusted for cell composition using estimates obtained from EstimateCellCounts.

## Discussion

In this manuscript, we have described and extensively evaluated a novel, iterative algorithm for assembling L-DMR libraries. Our objective was to present a methodology that can identify libraries that improve the prediction performance of CMD. Building off existing approaches [8, 13], IDOL involves the targeted curation of libraries whose constituent L-DMRs are selected on the basis of their collective ability to optimize the accuracy and minimize the prediction error associated with cell composition estimates obtained through CMD. The principal difference between IDOL and the assembly of existing L-DMR libraries is that IDOL makes use of a training data set consisting of samples with both WB DNA methylation signatures and immune profiling data as a means of calibrating the selection of L-DMRs. This in turn results in libraries that enhance the accuracy of CMD estimates and, as a consequence, improve the operating characteristics of EWAS, i.e., decreased false positive rate and increased statistical power.

In our application of IDOL to a training set, we assembled optimal L-DMR libraries across a range of possible library sizes (i.e., 100,200,…,800) in order to examine the relationship between library size and the accuracy of cell composition estimates. Although only modest differences in prediction performance were observed between the optimal libraries identified at each size considered, our results showed a trend toward diminishing prediction performance for sizes exceeding 500 L-DMRs. Though caution should be exercised when drawing conclusions on the basis of a single analysis, these results seem suggest that when it comes to assembling libraries for CMD, the quality of selected L-DMRs takes precedence over their quantity (i.e., library size). Despite being half the size, the prediction performance observed for the final IDOL library was on par with, and in many cases better than, EstimateCellCounts across both the training and independent testing sets. We hypothesized that the better performance associated with the IDOL library was a result of its ability to find libraries that better characterize the unique methylomic fingerprint of leukocyte subtypes. To examine this hypothesis, we compared each library in terms of how well it performed in discriminating each pair of cell types by computing the DSC. The results of this analysis showed that the IDOL library better discriminated 14 out of the 15 pairs of leukocyte subtypes, with significantly improved discrimination strength across the entire immune cell landscape. This observation is noteworthy in that it may suggest a framework for gauging the prognostic potential of DMR libraries in the absence of DNA methylation data sets with available immune profiling information, the “gold-standard” for assessing the prediction performance associated with different libraries. More importantly, our results serve to illustrate a key factor underlying the accuracy of cell composition estimates obtained via CMD, namely, that prediction accuracy is strongly related to a library’s ability to provide a powerful discrimination of the entire cellular landscape.

While the library used by EstimateCellCounts is a significant improvement over the TopANOVA approach for library assembly, it imposes an equal number of cell-specific L-DMRs for all cell types. In principle, this would be reasonable if cell types were mutually distinct from one another, however this is not the case for white blood cell types whose DNA methylation signatures are lineage-specific [3, 4, 13]. Because of the shared lineages of leukocyte subtypes, more or fewer L-DMRs might be needed for certain cell types depending on strength of their signal, within cell-type variability of those markers, and the lineage relationships between cell types. The data-driven approach for assembling libraries characteristic of IDOL indirectly addresses this issue by iteratively searching for the subset of L-DMRs that optimize the accuracy of CMD, with no a priori constraints on the number of cell-specific L-DMRs used in assembly of libraries. As demonstrated here, this approach resulted in a library that demonstrated highly accurate cell composition estimates in all data sets considered in our examination. Although the EstimateCellCounts library showed similar performance across the testing sets, the results of our simulation study and data applications showed that even modest improvements to the overall accuracy of cell fraction estimates results in non-negligible differences in the false positive rate and statistical power for EWAS. In particular, our simulation studies showed that when differences in the underlying cellular distribution between groups are large, tests of differential methylation adjusted for cell composition estimates obtained using the default EstimateCellCounts library can lead to an estimated 5 % inflation in the false positive rate compared to adjustments made using the IDOL library. On the scale of EWAS, which typically involve testing hundreds of thousands to millions of CpGs, this amounts to thousands to tens-of-thousands of CpGs being incorrectly classified as differentially methylated. Moreover, in both data applications cell fraction estimates obtained using the identified IDOL library demonstrated an improved ability to explain variation in whole-blood-derived DNA methylation signatures. This lead to increased statistical power, and as a result, fewer samples needed when cell composition correction was carried out using the IDOL library. Although the Liu and Hannum data applications revealed relatively minor differences in the number of samples needed between libraries, the corresponding cost-differential for a single study can be on the order of several thousand dollars considering the current cost of the Illumina HumanMethylation450 array (http://www.illumina.com/), a figure whose magnitude becomes substantial when taken across the entire spectrum of past, present, and future EWAS involving adjustments for cell composition via CMD.

Notwithstanding the potential of IDOL to identify L-DMR libraries that enhance the accuracy of cell type predictions obtained through CMD, this method is not without certain limitations. As IDOL does not include an evaluation of the prediction performance of all possible combinations of L-DMR libraries (i.e., $L \choose J^{\star }$), there is no guarantee that IDOL will arrive at globally optimal solutions. Because of the inherent computational burden that would be required to ensure global optimality in this case, we opted for a more computationally parsimonious approach wherein libraries are identified by sequentially selecting L-DMR subsets preferentially comprised of L-DMRs that were previously marked as beneficial to prediction accuracy in previous IDOL iterations. Our procedure resulted in a optimized library consisting of 300 L-DMRs, which compared favorably to existing L-DMR libraries and demonstrated excellent prediction performance in two independent testing data sets. Thus, while global optimality cannot be guaranteed our results are encouraging and provide assurance of the capacity of IDOL to identifying libraries that result in highly accurate estimates of cell composition.

It also deserves mentioning the the ability of IDOL to find libraries that better characterize the unique methylomic fingerprint of leukocyte subtypes comes at the expense of moderate increases in computational time compared to existing techniques for library assembly. Along these lines, the leave-one-out procedure employed in Step 4 of IDOL may unnecessarily contribute to slower convergence and thus increased computational demands. To this end, bootstrap resampling [30] as a substitute for our leave-one-out procedure may lead to faster convergence of IDOL and represents a potential opportunity for future enhancements to this methodology. Finally, while the applications presented herein targeted the HM450 BeadArray, we note that IDOL is generalizable to other platforms (i.e., whole-genome bisulfite sequencing, Illumina HumanMethylationEPIC BeadArray, ect.) provided that the reference methylomes for isolated leukocyte subtypes are available on those platforms. As interest in this area continues to grow, future studies should aim to compare the L-DMR library identified here to those identified from technologies with expanded coverage of the methylome.

## Conclusions

Motivated by the critical importance of accounting for cellular distribution when DNA methylation is assessed in heterogeneous tissue types [6–9, 20], along with the logistical and economic considerations that often render direct measurements of cell composition infeasible, our work fills a critical gap in the EWAS literature by reinforcing the importance of library assembly and its critical role in CMD. Further, we provide the epigenomics research community with a L-DMR library, optimized to improve the accuracy of cell distribution estimates in blood-derived biospecimens from human adults. Importantly, while motivated by the problem of deconvoluting the cellular mixture of whole blood, this research provides a framework for the systematic construction of DMR libraries in general, and represents a viable approach for library assembly for EWAS moving forward.

## Methods

In what follows, we begin by describing the DNA methylation array data sets used in this research as well as the steps implemented in their preprocessing and quality control. We next provide an overview of cell mixture deconvolution and the IDOL algorithm. Finally, we describe our application of IDOL, metrics employed for assessing and comparing cell type prediction performance, and finish by describing a data application for exploring the implications of cell composition adjustment in EWAS.

### Cell mixture reconstruction experiment

Purified granulocytes, monocytes, CD4T, CD8T, natural killer cells, and B cells from normal human subjects were purchased from AllCells LLC (Emeryville, CA). As described (http://www.allcells.com/normal-peripheral-blood/) ethical approval, including all consents and protocols, have been approved by an independent review board. Both positive and negative selection for relevant cell surface proteins was conducted by AllCells using antibodies conjugated to magnetic beads and protocols from Miltenyi Biotec, Inc. (Auburn, CA). DNA was extracted from purified blood leukocyte subtypes using the DNeasy blood and tissue kit (QIAGEN, Valencia CA) or the AllPrep DNA/RNA/Protein Mini Kit (QIAGEN) using previously described protocols [31]. DsDNA was quantified using a Qubit 3.0 fluorometer (Life Technologies). Following quantification, DNA extracted from purified leukocyte subtypes were mixed in predetermined proportions to reconstruct two distinct sets, consisting of *n*=6 samples each. The first set of reconstructed samples used mixtures of purified leukocyte subtype DNA in relatively equivalent proportions across the leukocyte subtypes, hereafter referred to as the MethodA samples. For the second set of six samples, the proportion of DNA for each leukocyte subtype were selected to resemble their relative fractions in the peripheral blood of normal human adult subjects (MethodB samples). All DNA samples were bisulfite modified using the Zymo EZ DNA Methylation kit (Irvine, CA) and epigenome-wide DNA methylation assessment was performed using the Illumina HumanMethylation450 array platform.

### Adult whole blood (WB) samples

An additional *n*=6 whole blood (WB) samples from disease-free adult donors with available immune cell profiling data from flow cytometry were purchased from AllCells LLC. Inclusion and exclusion criteria for donors as well as a statement describing the ethical approval of such samples can be found on the AllCells LLC webpage (http://www.allcells.com/normal-peripheral-blood/). We hereafter refer to this data set as the AdultMixed set. DNA extraction and bisulfite modification of the AdultMixed samples followed an identical protocol to that described above, with epigenome-wide DNA methylation profiling performed using the Illumina HumanMethylation450 array platform.

### Reference DNA methylomes for isolated leukocyte subtypes

To identify L-DMRs and as the basis of all applications of CMD, we used a publicly available data set (GEO Accession ID: GSE35069) consisting of epigenome-wide DNA methylation profiles for the same six leukocyte subtypes used in our reconstruction experiments. Further details concerning the study participants, purification of blood cell populations, and DNA extraction have been previously described [3].

### Additional DNA methylation data sets

In addition to the aforementioned data sets, we also made use of two of the largest publicly available blood-derived DNA methylation data sets currently available on Gene Expression Omnibus (Accession numbers: GSE42861 and GSE40279). Collectively, these two data sets consist of WB DNA methylation data on >1200 adult patients and were used here for the purpose of understanding the implications of cell mixture adjustment when mixture fractions were estimated using differing L-DMR libraries. The first data set (Liu) consisted of blood-derived DNA methylation data on 689 human subjects, including *n*=354 rheumatoid arthritis and *n*=335 non-diseased control patients [16]. The second data set (Hannum) included blood-derived DNA methylation data on 656 non-diseased adults, ranging in age from 19 to 101 years old [29]. For both data sets, epigenome-wide DNA methylation assessment was performed using the Illumina HumanMethylation450 array platform.

### Quality control and preprocessing of the DNA methylation data sets

For each of the data sets used in this research (Table 1), background subtraction and normalization utilizing various internal controls present on the Methylation450 BeadChip was conducted using the publicly available, *minfi* Bioconductor package (http://bioconductor.org). Every beta-value on the HumanMethylation450 array platform is accompanied with a detection *p*-value, representing the confidence that the signal intensities for that locus exceed the background defined by the negative control probes. To ensure high-quality methylation data, CpG loci having a sizable fraction (>25 %) of detection *p*-values above a predetermined threshold (detection *p*>10^−5^) were excluded from our analysis [20]. Also, we employed Subset quantile within array-normalization (SWAN) to adjust the beta-values of type 2 design probes into a statistical distribution characteristic of type 1 probes [32]. Finally, the presence of batch-effects, or technical sources of variability induced by plate and/or BeadChip, was assessed using principal components analysis (PCA) [33, 34]. Specifically, PCA was fit to the background subtracted and normalized methylation data and the top *S* principal components (*S* determined using a previously described approach [35]) were examined in terms of their association with plate and BeadChip. If plate and/or BeadChip was found to be significantly associated with any of the top *S* principal components (*p*<0.05), we applied ComBat [36, 37], an empirical Bayes batch-adjustment methodology that has become a standard pre-processing technique for array-based DNA methylation data [7, 20, 38].

**Table 1:** Summary of the data sets used in this research

Biospecimen	Name	Details	N	Training or testing	GEO ID	Description
Whole Blood (WB)	AdultMixed	Unfractioned peripheral blood leukocytes (PBL)	6	Training	GSE77797	DNAm profiling of WB samples collected from 6 different healthy adult donors.
	MethodA	Reconstructed cell mixtures	6	Testing	GSE77797	DNAm profiled in samples consisting of mixtures of CD4T, CD8T, NK, B cells, Monocytes, and Granulocytes, mixed predetermined proportions
	MethodB	Reconstructed cell mixtures	6	Testing	GSE77797	DNAm profiled in samples consisting of mixtures of CD4T, CD8T, NK, B cells, Monocytes, and Granulocytes, mixed predetermined proportions
	Liu	Unfractioned peripheral blood leukocytes (PBL)	689	Testing	GSE42861	DNAm profiled in WB samples collected from *n*=354 rheumatoid arthritis and *n*=335non-diseased control patients [16]
	Hannum	Unfractioned peripheral blood leukocytes (PBL)	656	Testing	GSE40279	DNAm profiled in WB samples collected from a total of 656 adults ranging in age from 19–101 years old [29]
Isolated cell types (Reference set)	Granulocytes (Gran)	Purified CD16+ cells	6	Both	GSE35069	DNAm profiling in purified cell types [3]
	Monocytes (Mono)	Purified CD14+ cells	6	Both	GSE35069	DNAm profiling in purified cell types [3]
	B cells (Bcell)	Purified CD19+ cells	6	Both	GSE35069	DNAm profiling in purified cell types [3]
	Natural Killer (NK)	Purified CD56+ cells	6	Both	GSE35069	DNAm profiling in purified cell types [3]
	CD8T	Purified CD3+CD8+ cells	6	Both	GSE35069	DNAm profiling in purified cell types [3]
	CD4T	Purified CD3+CD4+ cells	6	Both	GSE35069	DNAm profiling in purified cell types [3]

### Cell mixture deconvolution

To motivate the IDOL algorithm, we provide a brief description of CMD, referring interested readers to Houseman et al. (2012) for further details. Let $\phantom {\dot {i}\!}\mathbf {Y}_{i} = [ Y_{i1}, Y_{i2}, \ldots, Y_{{iJ}^{\star }}]$ represent the methylation beta-values across *J*^⋆^ CpG loci for target sample *i*. Further assume that for target sample *i*, DNA methylation was assessed over a heterogeneous cell population, comprised of a mixture of *K* underlying cell types whose proportions within sample *i* are given by: ***ω***_*i*_=[*ω*_*i*1_,*ω*_*i*2_,…,*ω*_*iK*_].

As first described in Houseman et al. (2012), the methylation signature of sample *i* is assumed to arise as a weighted mixture of the DNA methylation signature of each of the *K* underlying cell types:

(1) $$ \mathbb{E}[\mathbf{Y}_{i}] = \boldsymbol{\omega}_{i} \boldsymbol{\mu}', \qquad 0 \leq \omega_{ik} \leq 1 ~\text{and} \sum\limits_{k = 1}^{K} \omega_{ik} \leq 1   $$

where ***μ*** is a *J*^⋆^×*K* matrix of mean methylation beta-values whose rows represent the same ordering of the *J*^⋆^ CpGs in **Y**_*i*_ and whose columns represent the *K* distinct cell types. Thus, the (*jk*)^*th*^ element of ***μ*** represents the population mean beta-value for CpG *j* among cell type *k*. Following from Eq. (1), the objective of CMD involves estimating the mixture weights $\tilde {\boldsymbol {\omega }}_{i}$ that minimize:

(2) $$ \text{argmin}_{\omega_{i}}|| \mathbf{Y}_{i} - \boldsymbol{\omega}_{i}\boldsymbol{\mu}' ||^{2}   $$

subject to the aforementioned constraints on ***ω***_*i*_. Because ***μ*** is unobserved in practice, it is substituted with its sample mean **M**, estimated from one of several possible existing reference methylation data sets [3, 13].

The mainstay of CMD is that knowledge of the methylomic fingerprint of each cell type - represented by the column space of **M** - can be used to estimate their fractions within a sample consisting of a heterogenous mixture of those same cell types, **Y**_*i*_. As such, the ability to accurately estimate the underlying mixture composition of a given target sample depends entirely on the *J*^⋆^ CpGs (i.e., L-DMR library) being used as the basis of CMD. Ideally, L-DMR libraries should consist of CpGs whose methylation signature is maximally distinct across the *K* cell types and whose within-cell-type variation is minimal. Hence, efforts to improve the accuracy of cell composition estimates obtained through CMD should focus on identifying L-DMR libraries that satisfy the above criteria. To date, several strategies have been been proposed for assembling L-DMR libraries.

The first of such strategies involved assembling libraries using the *J*^⋆^≪*J* CpGs with the largest *F*-statistics computed from a series of ANOVA models fit to the DNA methylation profiles of purified isolated leukocyte cell types [13]. While reasonable in principle, using ANOVA *F*-statistics as the criteria for constructing libraries has the major limitation that libraries can become oversaturated with CpGs that discriminate certain leukocyte subsets (i.e., lymphoid- versus myeloid-cell-types), but lack sufficient signal for distinguishing closely related cell types. Recent attempts to address the limitations of the “ANOVA-based” strategy have instead used the top *L* hyper- and hypomethylated CpGs for each cell type, selected from a rank ordering of CpGs by their *t*-statistic computed from two-sample t-test comparisons of the methylation signature of each cell type against all other cell types [8]. This procedure is implemented in the Bioconductor package minfi:EstimateCellCounts [23], where, by default, the top 50 hyper- and hypomethylated CpGs for each cell type (i.e., CD4T, CD8T, NK, B cell, monocyte, granulocyte) are used to assemble the L-DMR library. By imposing an equal representation L-DMRs for each cell type, this strategy is much less prone to the oversaturation problem characteristic of the “ANOVA-based” approach; the net effect being improved discrimination of closely related cell types and as a result, more accurate estimates of cell composition.

### Algorithm for the optimal selection of L-DMRs

While the strategy for library assembly used by EstimateCellCounts is less susceptible to the types of issues that can arise when rank ordering CpGs using the *F*-statistic, it has several limitations that may curtail the accuracy of cell fraction estimates. In particular, because CpGs are selected irrespective of any evaluation of their contribution to the accuracy of cell fraction estimates, the EstimateCellCounts library may not necessarily coincide with the optimal set of CpGs for cell composition prediction. In addition, EstimateCellCounts uses a library that is comprised of an equal number of cell-specific L-DMRs (i.e, top 50 hyper- and hypomethylated cell-specific CpGs). While preventing scenarios where libraries are oversaturated with CpGs that only discriminate certain subsets of leukocytes, the assumption of an equal number of cell-specific CpGs may not necessarily correspond with optimal prediction accuracy. Finally, although using top hyper- and hypomethylated CpGs across each cell type for library assembly is an intuitive and sensible approach, it is possible that there exists a non-overlapping set of L-DMRs that outperform the top hyper- and hypomethylated CpGs in terms of prediction accuracy.

To address the limitations of existing L-DMR libraries, we propose IDOL, an algorithm that iteratively searches for libraries that improve the accuracy and precision of CMD. It is important to note that IDOL requires a training data set for calibrating the selection of optimal DMR libraries. For example, when focus is centered on identifying optimal DMR libraries for deconvoluting peripheral blood, training data sets should consist of samples with both WB DNA methylation signatures and direct measurements of the underlying cell distribution of those samples; i.e., CBC, FACS, etc. In what follows, we provide a detailed description of each step of the IDOL algorithm. Step 0: **Construction of the candidate L-DMR search space**

- Similar to [8], a series of two-sample *t*-tests (or similar methodology) are fit to the *J* arrayed CpGs and used to compare the mean methylation beta-values between each cell type against the mean beta-values computed across all other cell types.
- Identify the *L*/2 CpGs with the largest *t*-statistics and the *L*/2 CpGs with the smallest *t*-statistics for each of the *K* cell types, where *L* is a tuning parameter representing the number of cell-specific L-DMRs.
- Construct a set $\mathcal {Q}$, which consists of the *L* cell-specific L-DMRs identified in (b). Thus, $\mathcal {Q}$ is comprised of *P*=*L*×*K* putative L-DMRs, and represents the candidate search space for the subsequent steps of IDOL. It should be noted that there are trade-offs in the selection of *L*. Whereas large values of *L* broaden the candidate space in which to search for optimal L-DMR libraries, this comes at the expense of increased computational burden. Conversely, while small *L* results in lower computational costs, this comes with the risk missing potentially predictive L-DMRs due to a narrower candidate search space. Since the IDOL algorithm needs to be applied only when the reference methylomes for “new” cell types are added to those that currently exist (i.e., CD4T, CD8T, NK, Bcell, Monocytes, and Granulocytes), or if one wishes to identify optimized L-DMR libraries based on different technologies for interrogating the methylome (i.e., Illumina Human Methylation EPIC BeadArray, whole genome bisulfite sequencing, etc.), we advise users to select *L* to be arbitrarily large to ensure a broad enough candidate search space.
- In addition to pre-selecting *L*, the user also needs to pre-select *J*^⋆^≪*P*, representing the library size. It is important to note that special care should be given in the selection of *J*^⋆^, as the accuracy and precision of cell proportion estimates are sensitive to its specification [22]. We provide specific suggestions its selection at the end of this section.

Step 1: **Random assembly of L-DMR libraries**

- At iteration *ℓ*, *J*^⋆^ CpGs are randomly selected from $\mathcal {Q}$ with probability $\pi _{j}^{(\ell)}$, *j*=1,2,…,*P*. At iteration 0, every CpG among the *P* candidate L-DMRs has an equal chance of being selected, i.e., $\pi _{j}^{(0)} = 1/P$, $\forall j \in \mathcal {Q}$.
- Let $\mathcal {Q}^{(\ell)} \subset \mathcal {Q}$ represent the randomly assembled L-DMR library, comprised of the *J*^⋆^ randomly selected CpGs at iteration *ℓ*.

Step 2: **Cell composition estimation using randomly assembled library**

- Using the randomly assembled library $\mathcal {Q}^{(\ell)}$, apply CMD to a training set to obtain cell composition estimates: $\tilde {\boldsymbol {\omega }}_{i}$, where *i*=1,…,*N*_1_ and *N*_1_ represents the number of training samples.
- The resulting set of predictions are given as $\tilde {\boldsymbol {\Omega }} = [\tilde {\boldsymbol {\omega }}_{1}, \tilde {\boldsymbol {\omega }}_{2}, \ldots, \tilde {\boldsymbol {\omega }}_{N_{1}}]$, where $ 0 \leq \tilde {\boldsymbol {\omega }}_{i} \leq 1$ is a *K*×1 vector of the predicted cell proportions for training sample *i*. Further define $\tilde {\boldsymbol {\Omega }}_{k} = [\tilde {\boldsymbol {\omega }}_{1k}, \tilde {\boldsymbol {\omega }}_{2k}, \ldots, \tilde {\boldsymbol {\omega }}_{N_{1}k}]$ as the predicted proportions for cell type *k* across the *N*_1_ training samples.

Step 3: **Assessing the accuracy of cell composition estimates**: Given the strengths and limitations of purely relative and absolute measures for assessing prediction performance [39], we propose using both the *R*^2^ and root mean square error (*RMSE*) as the basis for our assessments. Let $\phantom {\dot {i}\!}\boldsymbol {\Omega } =\, [\boldsymbol {\omega }_{1}, \boldsymbol {\omega }_{2}, \ldots, \boldsymbol {\omega }_{N_{1}}]$ represent the observed cell proportions for the *N*_1_ target samples obtained via CBC, FACS, etc. The proportion of variation in the observed fraction of cell-type *k* (***Ω***_*k*_) explained by its predicted fraction ($\tilde {\boldsymbol {\Omega }}_{k}$) is computed as:

$${} {R_{k}^{2}} = 1 - \frac{\mathbf{1}_{N_{1}}' (\boldsymbol{\Omega}_{k} - \widehat{\boldsymbol{\Omega}}_{k})' (\boldsymbol{\Omega}_{k} - \widehat{\boldsymbol{\Omega}}_{k}) \mathbf{1}_{N_{1}}}</p><p class="noindent">{ \mathbf{1}_{N_{1}}' (\boldsymbol{\Omega}_{k} - \bar{\boldsymbol{\Omega}}_{k})' (\boldsymbol{\Omega}_{k} - \bar{\boldsymbol{\Omega}}_{k}) \mathbf{1}_{N_{1}}}, \qquad 0 \leq {R_{k}^{2}} \leq 1 $$

where $\bar {\boldsymbol {\Omega }}_{k} = \sum _{i = 1}^{N_{1}} \boldsymbol {\Omega }_{k}/N_{1}$ is an estimate of the mean observed fraction of cell-type *k* and $\widehat {\boldsymbol {\Omega }}_{k}$ represents the linear predictor obtained from regressing ***Ω***_*k*_ on $\tilde {\boldsymbol {\Omega }}_{k}$. In particular,

$$\widehat{\boldsymbol{\Omega}}_{k} = \widehat{\boldsymbol{\beta}}_{k} \tilde{\boldsymbol{\Omega}_{k}}</p><p class="noindent">$$

where, $\widehat {\boldsymbol {\beta }}_{k} = (\tilde {\boldsymbol {\Omega }_{k}}' \tilde {\boldsymbol {\Omega }_{k}})^{-1} \tilde {\boldsymbol {\Omega }_{k}}' \boldsymbol {\Omega }_{k}$. Thus, $\bar {R}^{2} = \frac {1}{K}\sum _{k = 1}^{K} {R_{k}^{2}}$ represents an estimate of the mean coefficient of determination across the *K* cell types. Additionally, the *RMSE* for cell type *k*=1,2,…,*K* is computed here using the following expression:

$$\begin{aligned}</p><p class="noindent">{} RMSE_{k} &= \sqrt{\frac{\mathbf{1}_{N_{1}}'(\boldsymbol{\Omega}_{k} - \tilde{\boldsymbol{\Omega}}_{k})' (\boldsymbol{\Omega}_{k} - \tilde{\boldsymbol{\Omega}}_{k})\mathbf{1}_{N_{1}}}{N_{1}}},\\ &\qquad 0 \leq RMSE_{k} < \infty \end{aligned} $$

with $\bar {M} = \frac {1}{K} \sum _{k = 1}^{K} RMSE_{k}$ representing an estimate of the mean *RMSE* across the *K* cell types. Given the above, IDOL seeks to find L-DMR libraries whose cell-type predictions minimize $\bar {M}$ and maximize $\bar {R}^{2}$. As described in further detail below, both $\bar {M}$ and $\bar {R}^{2}$ are used for determining the contribution of each CpG in $\mathcal {Q}^{(\ell)}$ on overall prediction performance. Step 4: **Leave-one out procedure**: In order to assess the individual contribution of each CpG in $\mathcal {Q}^{(\ell)}$, we implement the following leave-one-out procedure:

- Each of the *J*^∗^ CpGs contained in $\mathcal {Q}^{(\ell)}$ are iteratively removed to obtain the following sets $\mathcal {Q}^{(\ell)}_{-j}$, which include all CpGs in $\mathcal {Q}^{(\ell)}$, except for CpG *j*.
- **Steps 2–3** are repeated for each reduced library $\mathcal {Q}^{(\ell)}_{-j}$ and used to obtain ($\bar {M}_{-j}$, $\bar {R}_{-j}^{2})$; estimates of the overall *RMSE* and coefficient determination when CpG *j* is excluded from the L-DMR library. Conceptually, when $\bar {R}_{-j}^{2}$ is small relative to $\bar {R}^{2}$, this suggests that withholding CpG *j* from $\mathcal {Q}^{(\ell)}$ resulted in predictions that, on average, accounted for a smaller proportion of variation in the observed cell fractions. Conversely, when $\bar {R}_{-j}^{2} > \bar {R}^{2}$, withholding CpG *j* from $\mathcal {Q}^{(\ell)}$ resulted in predictions that accounted for a larger proportion of variation in the observed cell proportions. A similar argument holds for the relationship between $\bar {M}_{-j}$ and $\bar {M}$.
- From (b), it is clear that in subsequent IDOL iterations we would want to preferentially keep CpGs whose $\bar {M} - \bar {M}_{-j} < 0$ and $\bar {R}^{2} - \bar {R}_{-j}^{2} > 0$. This observation implies a framework for updating the selection probabilities of each CpG.

Step 5: **Updating selection probabilities**:

- Since *R*^2^ and *RMSE* are measured on different scales, we begin by normalizing both $\bar {M}_{-j}$ and $\bar {R}_{-j}^{2}$ to obtain *U*_−*j*_ and *V*_−*j*_, *j*=1,…*J*^∗^ respectively:
  $$ U_{-j} = \frac{\bar{M}_{-j} - \bar{M}}{\text{sd}\left(\bar{M}_{-j}\right)}, \qquad \qquad V_{-j} = \frac{\bar{R}^{2}_{-j} - \bar{R}^{2}}{\text{sd}\left(\bar{R}^{2}_{-j}\right)} $$
  where −*∞*<*U*_−*j*_<*∞* and −*∞*<*V*_−*j*_<*∞*.
- Noting that CpG *j* should be preferentially updated to have a larger probability of selection when both *U*_−*j*_ and −*V*_−*j*_ are large, we generate a composite measure by first converting (*U*_−*j*_,−*V*_−*j*_) from the Cartesian coordinate system to the polar coordinate system:
  $$\begin{array}{@{}rcl@{}} r_{-j} & = & \sqrt{\delta U^{2}_{-j} + (1-\delta)(-V_{-j})^{2}} \\ \theta_{-j} & = & \text{atan2}(-(1-\delta)V_{-j}, \text{} \delta U_{-j}) \end{array} $$
  where atan2 is a common variation of the arc tangent function, *r*_−*j*_ is the radial coordinate, *θ*_−*j*_ is the angular coordinate, and 0≤*δ*≤0 is a parameter that controls the balance between relative and absolute prediction performance. For example, when *δ*=1/2, a CpG’s influence on relative and absolute prediction performance receives equal weight. When *δ*→1 a CpG’s influence on absolute prediction performance receives more weight and when *δ*→0, a CpG’s influence on relative prediction performance receives more weight. The increment for modifying the selection probability of CpG *j* is given as:
  $$p_{-j} = r_{-j}\text{cos}(\theta_{-j} - \pi/4), \qquad -\infty \leq p_{-j} \leq \infty $$
  For the purpose of exposition, when *δ*=1/2, CpGs with the largest increment in selection probability (i.e., large *p*_−*j*_) are those with large *r*_−*j*_ and *θ*_−*j*_ close to *π*/4 radians (Fig. 2b,c). Conversely, CpGs with the largest decrease in selection probability (i.e., small *p*_−*j*_) are those with large *r*_−*j*_ and *θ*_−*j*_ close to 5*π*/4. When *p*_−*j*_≈0, this implies that either *r*_−*j*_ is small or *θ*_−*j*_ is close to (3*π*/4, −*π*/4) radians and suggests that withholding CpG *j* from $\mathcal {Q}^{(\ell)}$ is neither helpful nor detrimental to prediction performance. In these situations, the selection probability should remain unchanged.
- This brings us to the following procedure for updating selection probabilities,
  (3) $$ \pi_{j}^{(\ell + 1)} = \frac{\rho_{j}^{(\ell + 1)}}{\sum_{j \in \mathcal{Q}} \rho_{j}^{(\ell + 1)}}, \qquad 0 \leq \pi_{j}^{(\ell + 1)} \leq 1   $$
  where,
  (4) $$ \rho_{j}^{(\ell + 1)} = \Bigg\{ \begin{array}{ll} \pi_{j}^{(\ell)}\text{expit}(p_{-j}) + \pi_{j}^{(\ell)}/2 & \quad \text{if} j \in \mathcal{Q}^{(\ell)} \\ \pi_{j}^{(\ell)} & \quad \text{if} j \not \in \mathcal{Q}^{(\ell)} \end{array}   $$
  and expit is the inverse-logit function, i.e., expit(*x*)= exp(*x*)/(1+ exp(*x*)). Thus, selection probabilities for each $j \in \mathcal {Q}^{(\ell)}$ are modified based on how beneficial/not beneficial each CpGs was determined to be in the presence of the remaining *J*^⋆^−1 CpGs. As noted from Eqs. (3 and 4), the probability of selection is unchanged for CpGs $j \not \in \mathcal {Q}^{(\ell)}$ as well as for CpGs where *p*_−*j*_≈0.

Step 6: **Continue Iteration**: Using the updated probabilities, $\pi _{j}^{(\ell + 1)}$, *j*=1,…,*P*, repeat steps 1-5. The final solution consists of the library comprised of the *J*^⋆^ CpGs with the largest selection probabilities (Fig. 2).

As previously described, because the accuracy and precision of cell proportion estimates are sensitive to the specification of *J*^⋆^, special treatment should be given towards its selection. Although computationally demanding, our strategy for determining *J*^⋆^ involves fitting IDOL across a range of possible values for *J*^⋆^, (i.e., *J*^⋆^={50,100,200,…}) followed by a comparison of prediction performance across each of the specified values. Under such a framework, we select the smallest value of *J*^⋆^ upon which the gains in prediction performance for increasing values of *J*^⋆^ is minimal, (i.e., within some predetermined tolerance of the performance metrics).

### Application and assessment of IDOL

#### Training the L-DMR selection algorithm

To examine the robustness of IDOL, we employed a training and testing procedure and benchmarked theprediction performance of the library identified by IDOL against the widely used EstimateCellCounts function in the *minfi* Bioconductor package. Specifically, we first applied IDOL to the AdultMixed samples (Training Set) to identify “optimal” L-DMR libraries for deconvoluting the cell distribution of whole blood. As previously described, the AdultMixed samples consisted of both flow cytometric measurements and whole blood DNA methylation data derived from the same set of biospecimens used for flow cytometry. To examine the sensitivity of prediction performance based on the number of L-DMRs used for deconvoluting cellular mixture, we applied IDOL to the training samples assuming a range of possible values for *J*^⋆^, specifically assuming *J*^⋆^={100,200,300,400,500,600,700,800}. The final selection of *J*^⋆^ and the representative IDOL library used in our subsequent validation analyses was chosen to be the value *J*^⋆^ that resulted best prediction performance in the training set. Finally, in training the IDOL algorithm, selection probabilities of putative L-DMRs were updated assuming equal weights in terms of their contribution to relative and absolute prediction performance, i.e., *δ*=1.

Following the application of IDOL to the training set, we next examined the overlap between the “optimal” IDOL library and the 600 L-DMRs currently used by EstimateCellCounts. In order to comprehend the nature of the difference between these libraries and how such differences might influence their propensity for accurate cell fraction estimates, we computed the dispersion separability criterion (DSC). The DSC was initially developed as a metric for quantifying the extent of batch effects in ’omic data sets, and is computed as the ratio of the average distance between batch centroids and the global mean (*D*_between_) and the average distance between samples belonging to the same batch (*D*_within_). Larger values of DSC indicate greater dispersion between batches than within batches; i.e., samples within batches are more homogeneous compared to samples in different batches. In the same way, the DSC can be used for quantifying the dispersion between and within specific leukocyte subtypes based on a given set of L-DMRs, substituting batch with cell-type identity of a given sample. Using reference DNA methylation data profiled across the six major leukocyte components of whole blood [3], we computed the overall DSC and the DSC between each pair of cell types (i.e., CD4T vs CD8T, CD4T vs NK, etc.) using both the “optimal” IDOL library and the EstimateCellCounts library. Equation 5 provides the DSC formula for pairwise comparisons, where (*r,s*) denotes the two cell types being compared, $D^{(r,\,s)}_{\text {between}}$ represents the the average distance between cell type centroids and the global mean, and $D^{(r,\,s)}_{\text {within}}$ represents average distance between samples of the same cell type.

(5) $$ \begin{aligned} {} DSC^{(r,\,s)} &= \frac{D^{(r,\,s)}_{\text{between}}}{D^{(r,\,s)}_{\text{within}}}, (r,s) \in \left\{(1,2),\ldots,\right.\\ &\qquad \left.(1,K), \ldots, (K-1,K)\right\} \end{aligned}  $$

In order to assess which L-DMR library exhibited better performance at discriminating specific pairs of cell types (i.e., (*r,s*)), we computed the difference between DSCs calculated from the IDOL and EstimateCellCounts libraries (Eq. 6).

(6) $$ \Delta^{(r,\,s)} = DSC_{\text{IDOL}}^{(r,\,s)} - DSC_{\text{EstimateCellCounts}}^{(r,\,s)}   $$

Based on Eq. 6, *Δ*^(*r*, *s*)^=0 signifies no difference between the IDOL and EstimateCellCounts libraries for discriminating cell types *r* and *s*, whereas large positive or negative values of *Δ*^(*r*, *s*)^ signify improved discrimination associated with the IDOL library (former) or the EstimateCellCounts library (latter). To test the hypothesis that *Δ*^(*r*, *s*)^=0, we conducted a non-parametric, randomization-based test. Specifically, *p*-values were computed by comparing the observed DSC differences to the empirical null distribution, generated through repeated random permutations of the data. Randomization-based *p*-values less than 0.05 were treated as statistically significant.

#### Independent validation of the optimal L-DMR set

To validate IDOL, we applied CMD to two independent test sets (MethodA and MethodB sets) using the optimal IDOL library identified in the training set. Our choice to use the MethodA and MethodB samples as our testing sets was motivated by the fact that the samples in both sets were obtained by mixing leukocyte subtype-specific DNA in known, predetermined proportions. Thus, for a given sample, the underlying leukocyte fractions are known with high confidence and are likely less prone to the measurement error associated cell sorting/counting techniques. As such, the MethodA and MethodB sets represent ideal data sets for validating the prognostic performance of the optimal L-DMR library identified in the training set.

To assess the performance of our cell type predictions, we estimated the proportion of variation of the known, reconstructed mixture fractions explained by our cell type predictions (i.e., *R*^2^) as well as the average deviation between the reconstructed mixture fractions and our predictions (i.e., *RMSE*). *R*^2^ and *RMSE* were computed for each cell type individually, across all testing samples and within each testing set separately. The rationale for latter was to examine the robustness of the IDOL library when the underlying cellular landscape differed (see Section ‘Cell mixture reconstruction experiment’ for further details on the MethodA and MethodB reconstruction experiment). As an additional comparison and to benchmark the performance of the IDOL library for accurately deconvoluting cellular mixture, we also applied the minfi:EstimateCellCounts function (using its default options). In a similar manner, cell-specific *R*^2^ and *RMSE* were computed based on the cell type predictions obtained from EstimateCellCounts, both within and across the two MethodA and MethodB sets.

### Simulation study comparing false discovery rates (FDR) across different cell composition adjustment techniques

To understand the consequences of prediction error in cell fraction estimates for EWAS, we conducted a simulation study to compare the false discovery rate (FDR) when different strategies for cell composition adjustment were employed. For our simulations, we assumed simplistic study design that, typical of many EWAS, focused on the identification of differentially methylated CpG sites between two groups, i.e, case/control comparison. To determine if the relationship between cell composition adjustment method and FDR was sensitive to the study sample size (i.e., *n*=*n*_1_+*n*_2_), we conducted separate simulations that ranged from small/moderately sized studies (i.e., *n*_1_,*n*_2_={50,100}) to large studies (i.e., *n*_1_,*n*_2_={250,500}). In addition to varying the sample sizes of each group, we also examined the relationship between FDR as a function of the dissimilarity in the true, simulated cell distribution between the two groups.

To motivate the design of our simulation study, we assumed that the methylation beta-value for CpG *j* among target sample *i*, *Y*_*ij*_, follows a beta-distribution with expectation and variance given by: ***ω***_*i*_***μ****j*′ and $\frac {(1-\boldsymbol {\omega }_{i} \boldsymbol {\mu }_{j}') \boldsymbol {\omega }_{i} \boldsymbol {\mu }_{j}'}{1 + \phi _{j}}$, respectively. As previously, ***ω***_*i*_ is vector of length *K* representing the true underlying cell fractions for sample *i*, ***μ***_*j*_ is a vector whose elements represent the population mean beta-values for CpG *j* across the *K* cell types, and *ϕ*_*j*_>0 is the unobserved dispersion parameter for CpG *j*. Letting *X*_*i*_ denote the group membership for sample *i*, many EWAS involve fitting regression models that have the following form:

(7) $$ \begin{aligned} {} Y_{ij} &= \alpha_{0j} + \alpha_{1j}X_{i} + \sum_{k = 1}^{K-1} \gamma_{kj} \omega_{ik} + \epsilon_{ij},\\& \qquad \mathbb{E}[\epsilon_{ij}] = 0~ \text{and~} \mathbb{V}[\!\epsilon_{ij}] = {\sigma^{2}_{j}} \end{aligned}  $$

where the term $\sum _{k = 1}^{K-1} \gamma _{kj} {\omega }_{ik}$ is introduced to control for cell composition differences across subjects and *ε*_*ij*_ captures the remaining variation in methylation after taking group status and cellular composition into account. In the above regression model, interest is typically centered on testing the hypothesis of no difference in DNA methylation levels between groups, i.e., *α*_1*j*_=0. However, in practice *ω*_*ik*_ is unknown and typically substituted with its estimate $\tilde {\omega }_{ik}$, obtained for example by CMD [13]. Since $\tilde {\omega }_{ik}$ is an estimate and therefore subject to uncertainty, tests of hypothesis and confidence intervals based on model 7 can become unreliable and prone to inflated Type 1 and 2 error rates.

To examine how cell type prediction errors associated with the IDOL and EstimateCellCounts libraries impact the FDR for testing *α*_1*j*_, we first estimated the uncertainty of cell fraction predictions for each method by squaring the *RMSE*s computed across the MethodA and MethodB testing sets to obtain the mean squared prediction errors (*MSPE*s):

(8) $$ \begin{aligned} {} \widehat{\tau}^{2}_{kl}& = MSPE_{kl} = RMSE^{2}_{kl} = \sqrt{\frac{1}{N} \sum_{i = 1}^{N} (\omega_{ik} - \tilde{\omega}_{ikl})^{2}},\\ &\qquad k = 1,2,\ldots K \end{aligned}  $$

where *l* is an index representing the library used for CMD (i.e., *l*={EstimateCellCounts, IDOL }) and *N* represents the total sample size for the testing data (i.e., *N*=12 for the MethodA and MethodB sets). After obtaining estimates of precision, $\widehat {\tau }^{2}_{kl}$, we implemented the following seven steps in our simulation study:

- Randomly sample *G*=10,000 CpGs from the Illumina HumanMethylation450 array.
- Estimate the dispersion parameter within the combined testing sets for each of the *G* randomly selected CpGs, $\widehat {\phi }_{g}$, *g*=1,2,…*G*. In addition, using the reference leukocyte methylation data [3], estimate cell-specific mean methylation beta-values for each of the *G* CpGs, *m*_*kg*_, *g*=1,2,…*G* and *k*=1,2,…*K*. Parameter estimation was carried out using method of moments estimation.
- Randomly generate the cell distribution for groups 1 and 2.
  - For group 1, simulate the cell distribution, ***ω***^(1)^, from a Dirichlet distribution with concentration parameters, $\boldsymbol {\nu }^{(1)} = [\nu ^{(1)}_{1}, \nu ^{(1)}_{2}, \ldots \nu ^{(1)}_{K}]$.
  - For group 2, simulate the cell distribution, ***ω***^(2)^, from a Dirichlet distribution with concentration parameters, $\boldsymbol {\nu }^{(2)} = [\nu ^{(2)}_{1}, \nu ^{(2)}_{2}, \ldots \nu ^{(2)}_{K}]$.
- For both groups, simulate methylation beta-values for each of the *G* CpGs from a beta-distribution.
  - For each of the *n*_1_ samples in group 1, randomly sample beta-values $Y^{(1)}_{ig}$ from a beta-distribution with mean ***ω***^(1)^**m***g*′ and variance $\frac {(1-\boldsymbol {\omega }^{(1)} \boldsymbol {m}_{g}') \boldsymbol {\omega }^{(1)} \boldsymbol {m}_{g}'}{1 + \widehat {\phi }_{g}}$.
  - For each of the *n*_2_ samples in group 2, randomly sample beta-values $Y^{(2)}_{ig}$ from a beta-distribution with mean ***ω***^(2)^**m***g*′ and variance $\frac {(1-\boldsymbol {\omega }^{(2)} \boldsymbol {m}_{g}') \boldsymbol {\omega }^{(2)} \boldsymbol {m}_{g}'}{1 + \widehat {\phi }_{g}}$.
- Randomly sample cell type predictions for each sample (i.e., $\tilde {\boldsymbol {\omega }}^{(1)}_{il}$ and $\tilde {\boldsymbol {\omega }}^{(2)}_{il}$) based using the cell-specific uncertainty estimates (Eq. 8) associated with the EstimateCellCounts and Optimized L-DMR methods.
  - For each of the *n*_1_ samples in group 1, obtain $\tilde {\boldsymbol {\omega }}^{(1)}_{il}$ by randomly sampling from a multivariate normal distribution with mean ***ω***^(1)^ and variance-covariance, $\Sigma ^{(1)}_{l} = \text {diag}(\widehat {\tau }^{2}_{kl})$, *k*=1,2,…*K* and *l*= (EstimateCellCounts or IDOL).
  - For each of the *n*_2_ samples in group 2, obtain $\tilde {\boldsymbol {\omega }}^{(2)}_{il}$ by randomly sampling from a multivariate normal distribution with mean ***ω***^(2)^ and variance-covariance, $\Sigma ^{(2)}_{l} = \text {diag}(\widehat {\tau }^{2}_{kl})$, *k*=1,2,…*K* and *l*= (EstimateCellCounts or IDOL).
- Fit model 7 to each of the *G* CpGs, adjusting for cell composition using the cell type predictions generated in Step 4. Based on the model fit, test the hypothesis, *H*_0_:*α*_1*g*_=0, for *g*=1,2,…,*G*.
- Calculate the FDR for each method assuming a nominal p-value cutoff of 0.05 for declaring CpGs as statistically significant.
- Repeat steps 1-7.

Since the beta-values for groups 1 and 2 were simulated assuming no group effect (i.e., assuming *α*_1*g*_=0), the methylation profile for groups 1 and 2 differ only with respect to the dissimilarity in the cell composition between groups, **Dissimilarity**:=||***ω***^(1)^−***ω***^(2)^||. Thus, rejections of the hypothesis *H*_0_:*α*_1*g*_=0 based on fitting model 7 to the simulated data signify Type 1 errors. As a measure to ensure that the FDR was correctly controlled at 5 % in models that controlled for the true, simulated cell distributions, we also augmented our simulation study with models that included adjustment for terms, ***ω***^(1)^ and ***ω***^(2)^.

### Data application for exploring the implications of cell composition adjustment in EWAS

To further understand the implications of cell type prediction methodology for EWAS (particularly, those using blood-derived DNA methylation data), we made use of two of the largest, publicly available, blood-derived DNA methylation data sets [16, 29]. Our analysis of these data sets was aimed at addressing two different but related questions: (*i*) which cell prediction methodology performed better at explaining variation in DNA methylation within each data set and (*ii*) how do differences in the variation being explained relate to the statistical power of such studies. To address these questions, we began by applying CMD [13] for estimating the immune cell composition of the samples in the Liu and Hannum data sets. CMD was applied using both the EstimateCellCounts (default settings) and the optimal IDOL library, giving rise to two sets of cell type predictions for each of the two data sets. For each data set, linear regression models were fit to the *J* CpG loci independently, modeling methylation beta-values as the response against the predicted cell distribution. Based on the fitted regression models, we estimated the variation in methylation unaccounted for by our estimates of cell mixture (i.e., residual variance) as well as the proportion of variation in methylation explained by cell mixture estimates: $R^{2}_{jl}$, *j*=1,2,…,*J* and *l*={EstimateCellCounts, IDOL}). Using these estimates, the difference in *R*^2^ between models adjusted for cell mixture using the optimal IDOL library versus EstimateCellCounts were computed for each of the *J* CpGs; i.e., $D_{j} = R^{2}_{j, \text {IDOL}} - R^{2}_{j, \text {EstimateCellCounts}}$.

To answer the first of our questions - which cell prediction methodology performs better at explaining variation in DNA methylation? - we computed the proportion of CpG loci where the IDOL library resulted in more variation in DNA methylation explained compared to EstimateCellCounts, i.e., $\frac {1}{J} \sum _{j = 1}^{J} \mathbb {I}(D_{j} > 0)$. To assess whether the observed proportion was greater than would be expected at random, we employed a non-parametric randomized-based test with a p-value cutoff of 0.05 to determine statistical significance.

We next sought to compare the impact of different L-DMR libraries on the statistical power of EWAS. Similar to our simulation study (Section ‘Simulation study comparing false discovery rates (FDR) across different cell composition adjustment techniques’), we assumed a simple study design that was aimed at identifying differences in the mean methylation levels between two groups. Using the residual variance estimates obtained above, we computed the sample size required for identifying differences in the mean methylation levels between groups that ranged from 0.01 to 0.05 on the beta-value scale. For our sample size estimates, we assumed a two-sample *t*-test, 80 % power, and Bonferroni corrected type 1 error rate (i.e, *α*/400,000) to account for issue of multiple testing encountered in EWAS. Within both the Liu and Hannum data sets, we randomly sampled the residual variance estimates for 1000 CpG loci obtained for each cell mixture correction methodology and computed the sample size needed for detecting a difference in mean methylation based on the previously mentioned assumptions. For a given difference in mean methylation, the sample size estimates based on the 1000 randomly sampled residual variance estimates were summarized by computing the mean, which formed the basis for our comparisons.

To highlight the economic implications of our findings, we also estimated the cost-differential for EWAS when cell mixture correction was carried out using the IDOL library versus EstimateCellCounts based on our estimates of the required sample sizes for each methodology. Cost-differential estimates were obtained by using the current per-sample cost of the Illumina HumanMethylation450 array of approximately 300 US dollars (http://www.illumina.com/).

## Additional files

Additional file 1
**Table S1.** Summary of cell distribution measurements/cell mixture adjustment, among random sample of EWAS published in 2015. (XLSX 11 kb)

Additional file 2
**Figure S1.** Bland-Altman plots constructed from cell fraction estimates of the *n*=6 AdultMixed samples obtained using the (**a**) TopANOVA, (**b**) EstimateCellCounts, and (**c**) IDOL optimized L-DMR libraries. (PDF 344 kb)

Additional file 3
**Table S2.** CpG-specific annotations for the candidate L-DMR library used in the IDOL algorithm. (XLSX 241 kb)

Additional file 4
**Figure S2.** Performance metrics for the optimal libraries identified by applying IDOL to the training set. (**a**) Average R-squared (computed across cell types) in the training set based on the optimal libraries at each assumed library size. (**a**) Average RMSE (computed across cell types) in the training set based on the optimal libraries at each assumed library size. (PDF 324 kb)

Additional file 5
**Table S3.** CpG-specific annotations for optimal L-DMR library identified by the IDOL algorithm. (XLSX 77 kb)

Additional file 6
**Table S4.** Comparison of prediction performance between the optimal IDOL library and EstimateCellCounts within both testing sets. (XLSX 50 kb)

Additional file 7
**Figure S3.** Bland-Altman plots constructed from cell fraction estimates of the *n*=6 MethodA reconstruction samples obtained using the (**a**) TopANOVA, (**b**) EstimateCellCounts, and (**c**) IDOL optimized L-DMR libraries. (PDF 306 kb)

Additional file 8
**Figure S4.** Bland-Altman plots constructed from cell fraction estimates of the *n*=6 MethodB reconstruction samples obtained using the (**a**) TopANOVA, (**b**) EstimateCellCounts, and (**c**) IDOL optimized L-DMR libraries. (PDF 282 kb)

Additional file 9
**Figure S5.** Cell type prediction performance (*R* ^2^ (top) and *RMSE* (bottom)) as a function of each cell type’s mean observed mixture fraction in the MethodA reconstruction samples. (PDF 698 kb)

## Abbreviations

EWAS: epigenome-wide association studies
CMD: cell mixture deconvolution
L-DMR: leukocyte differentially methylated region
WB: whole blood
FDR: false discovery rate
DSC: dispersion separability criterion
RMSE: root mean squared error
CBC: complete blood cell counts
FACS: fluorescence-activated cell sorting
HM450: Illumina HumanMethylation450 BeadArray

## References

[1] Rakyan VK, Down TA, Balding DJ, Beck S (2011). Epigenome-wide association studies for common human diseases. Nat Rev Genet.

[2] Adalsteinsson BT, Gudnason H, Aspelund T, Harris TB, Launer LJ, Eiriksdottir G, Smith AV, Gudnason V (2012). Heterogeneity in white blood cells has potential to confound dna methylation measurements. PLoS ONE.

[3] Reinius LE, Acevedo N, Joerink M, Pershagen G, Dahln SE, Greco D, Sderhll C, Scheynius A, Kere J (2012). Differential dna methylation in purified human blood cells: implications for cell lineage and studies on disease susceptibility. PLoS ONE.

[4] Koestler DC, Marsit CJ, Christensen BC, Accomando W, Langevin SM, Houseman EA, Nelson HH, Karagas MR, Wiencke JK, Kelsey KT (2012). Peripheral blood immune cell methylation profiles are associated with nonhematopoietic cancers. Cancer Epidemiol Biomarkers Prev.

[5] Lam LL, Emberly E, Fraser HB, Neumann SM, Chen E, Miller GE, Kobor MS (2012). Factors underlying variable dna methylation in a human community cohort. Proc Natl Acad Sci U S A.

[6] Houseman EA, Kim S, Kelsey KT, Wiencke JK (2015). Dna methylation in whole blood: Uses and challenges. Curr Environ Health Rep.

[7] Michels KB, Binder AM, Dedeurwaerder S, Epstein CB, Greally JM, Gut I, Houseman EA, Izzi B, Kelsey KT, Meissner A, Milosavljevic A, Siegmund KD, Bock C, Irizarry RA (2013). Recommendations for the design and analysis of epigenome-wide association studies. Nat Methods.

[8] Jaffe AE, Irizarry RA (2014). Accounting for cellular heterogeneity is critical in epigenome-wide association studies. Genome Biol.

[9] Liang L, Cookson WOC (2014). Grasping nettles: cellular heterogeneity and other confounders in epigenome-wide association studies. Hum Mol Genet.

[10] Reynolds LM, Taylor JR, Ding J, Lohman K, Johnson C, Siscovick D, Burke G, Post W, Shea S, Jacobs DRJr, Stunnenberg H, Kritchevsky SB, Hoeschele I, McCall CE, Herrington DM, Tracy RP, Liu Y (2014). Age-related variations in the methylome associated with gene expression in human monocytes and t cells. Nat Commun.

[11] Gunawardhana LP, Gibson PG, Simpson JL, Benton MC, Lea RA, Baines KJ (2014). Characteristic dna methylation profiles in peripheral blood monocytes are associated with inflammatory phenotypes of asthma. Epigenetics.

[12] Marioni RE, Shah S, McRae AF, Ritchie SJ, Muniz-Terrera G, Harris SE, Gibson J, Redmond P, Cox SR, Pattie A, Corley J, Taylor A, Murphy L, Starr JM, Horvath S, Visscher PM, Wray NR, Deary IJ. The epigenetic clock is correlated with physical and cognitive fitness in the lothian birth cohort 1936. Int J Epidemiol. 2015. doi:10.1093/ije/dyu277. 10.1093/ije/dyu277 PMC4588858 25617346

[13] Houseman EA, Accomando WP, Koestler DC, Christensen BC, Marsit CJ, Nelson HH, Wiencke JK, Kelsey KT (2012). Dna methylation arrays as surrogate measures of cell mixture distribution. BMC Bioinformatics.

[14] Zou J, Lippert C, Heckerman D, Aryee M, Listgarten J (2014). Epigenome-wide association studies without the need for cell-type composition. Nat Methods.

[15] Houseman EA, Molitor J, Marsit CJ (2014). Reference-free cell mixture adjustments in analysis of dna methylation data. Bioinformatics.

[16] Liu Y, Aryee MJ, Padyukov L, Fallin MD, Hesselberg E, Runarsson A, Reinius L, Acevedo N, Taub M, Ronninger M, Shchetynsky K, Scheynius A, Kere J, Alfredsson L, Klareskog L, Ekstrm TJ, Feinberg AP (2013). Epigenome-wide association data implicate dna methylation as an intermediary of genetic risk in rheumatoid arthritis. Nat Biotechnol.

[17] Koestler DC, Chalise P, Cicek MS, Cunningham JM, Armasu S, Larson MC, Chien J, Block M, Kalli KR, Sellers TA, Fridley BL, Goode EL (2014). Integrative genomic analysis identifies epigenetic marks that mediate genetic risk for epithelial ovarian cancer. BMC Med Genomics.

[18] Koestler DC, Avissar-Whiting M, Houseman EA, Karagas MR, Marsit CJ (2013). Differential dna methylation in umbilical cord blood of infants exposed to low levels of arsenic in utero. Environ Health Perspect.

[19] Liang L, Willis-Owen SAG, Laprise C, Wong KCC, Davies GA, Hudson TJ, Binia A, Hopkin JM, Yang IV, Grundberg E, Busche S, Hudson M, Rnnblom L, Pastinen TM, Schwartz DA, Lathrop GM, Moffatt MF, Cookson WOCM (2015). An epigenome-wide association study of total serum immunoglobulin e concentration. Nature.

[20] Wilhelm-Benartzi CS, Koestler DC, Karagas MR, Flanagan JM, Christensen BC, Kelsey KT, Marsit CJ, Houseman EA, Brown R (2013). Review of processing and analysis methods for dna methylation array data. Br J Cancer.

[21] Jones MJ, Islam SA, Edgar RD, Kobor MS. Adjusting for cell type composition in dna methylation data using a regression-based approach. Methods Mol Biol. 2015. doi:10.1007/7651_2015_262. 10.1007/7651_2015_262 26126446

[22] Koestler DC, Christensen B, Karagas MR, Marsit CJ, Langevin SM, Kelsey KT, Wiencke JK, Houseman EA (2013). Blood-based profiles of dna methylation predict the underlying distribution of cell types: a validation analysis. Epigenetics.

[23] Aryee MJ, Jaffe AE, Corrada-Bravo H, Ladd-Acosta C, Feinberg AP, Hansen KD, Irizarry RA (2014). Minfi: a flexible and comprehensive bioconductor package for the analysis of infinium dna methylation microarrays. Bioinformatics.

[24] Hertz J, Krogh A, Palmer G. Introduction to the Theory of Computation: Addison-Wesley; 1993.

[25] (McClatchey KD, editor.)2002. Clinical Laboratory Medicine. Lippincott Williams & Wilkins.

[26] Flanagan JM, Brook MN, Orr N, Tomczyk K, Coulson P, Fletcher O, Jones ME, Schoemaker MJ, Ashworth A, Swerdlow A, Brown R, Garcia-Closas M (2015). Temporal stability and determinants of white blood cell dna methylation in the breakthrough generations study. Cancer Epidemiol Biomarkers Prev.

[27] Marioni RE, Shah S, McRae AF, Chen BH, Colicino E, Harris SE, Gibson J, Henders AK, Redmond P, Cox SR, Pattie A, Corley J, Murphy L, Martin NG, Montgomery GW, Feinberg AP, Fallin MD, Multhaup ML, Jaffe AE, Joehanes R, Schwartz J, Just AC, Lunetta KL, Murabito JM, Starr JM, Horvath S, Baccarelli AA, Levy D, Visscher PM, Wray NR, Deary IJ (2015). Dna methylation age of blood predicts all-cause mortality in later life. Genome Biol.

[28] Demerath EW, Guan W, Grove ML, Aslibekyan S, Mendelson M, Zhou YH, Hedman K, Sandling JK, Li LA, Irvin MR, Zhi D, Deloukas P, Liang L, Liu C, Bressler J, Spector TD, North K, Li Y, Absher DM, Levy D, Arnett DK, Fornage M, Pankow JS, Boerwinkle E (2015). Epigenome-wide association study (ewas) of bmi, bmi change and waist circumference in african american adults identifies multiple replicated loci. Hum Mol Genet.

[29] Hannum G, Guinney J, Zhao L, Zhang L, Hughes G, Sadda S, Klotzle B, Bibikova M, Fan JB, Gao Y, Deconde R, Chen M, Rajapakse I, Friend S, Ideker T, Zhang K (2013). Genome-wide methylation profiles reveal quantitative views of human aging rates. Mol Cell.

[30] Efron B, Tibshirani R. An Introduction to the Bootstrap: Chapman & Hall/CRC; 1993.

[31] Accomando WP, Wiencke JK, Houseman EA, Nelson HH, Kelsey KT (2014). Quantitative reconstruction of leukocyte subsets using dna methylation. Genome Biol.

[32] Maksimovic J, Gordon L, Oshlack A (2012). Swan: Subset-quantile within array normalization for illumina infinium humanmethylation450 beadchips. Genome Biol.

[33] Harper KN, Peters BA, Gamble MV (2013). Batch effects and pathway analysis: two potential perils in cancer studies involving dna methylation array analysis. Cancer Epidemiol Biomarkers Prev.

[34] Teschendorff AE, Menon U, Gentry-Maharaj A, Ramus SJ, Gayther SA, Apostolidou S, Jones A, Lechner M, Beck S, Jacobs IJ, Widschwendter M (2009). An epigenetic signature in peripheral blood predicts active ovarian cancer. PLoS ONE.

[35] Teschendorff AE, Zhuang J, Widschwendter M (2011). Independent surrogate variable analysis to deconvolve confounding factors in large-scale microarray profiling studies. Bioinformatics.

[36] Johnson WE, Li C, Rabinovic A (2007). Adjusting batch effects in microarray expression data using empirical bayes methods. Biostatistics.

[37] Leek JT, Johnson WE, Parker HS, Jaffe AE, Storey JD (2012). The sva package for removing batch effects and other unwanted variation in high-throughput experiments. Bioinformatics.

[38] Bock C (2012). Analysing and interpreting dna methylation data. Nat Rev Genet.

[39] Steyerberg EW, Vickers AJ, Cook NR, Gerds T, Gonen M, Obuchowski N, Pencina MJ, Kattan MW (2010). Assessing the performance of prediction models: a framework for traditional and novel measures. Epidemiology.

