# Supplementary material for: Improving cell mixture deconvolution by identifying optimal DNA methylation libraries (IDOL)
Source: BMC Bioinformatics. 2016 Mar 8;17:120. doi: 10.1186/s12859-016-0943-7 (PMC4782368; doi:10.1186/s12859-016-0943-7)
Supplement: Additional file 2 — Figure S1. Bland-Altman plots constructed from cell fraction estimates of the n=6 AdultMixed samples obtained using the (a) TopANOVA, (b) EstimateCellCounts, and (c) IDOL optimized L-DMR libraries. (PDF 344 kb) [file 12859_2016_943_MOESM2_ESM.pdf]

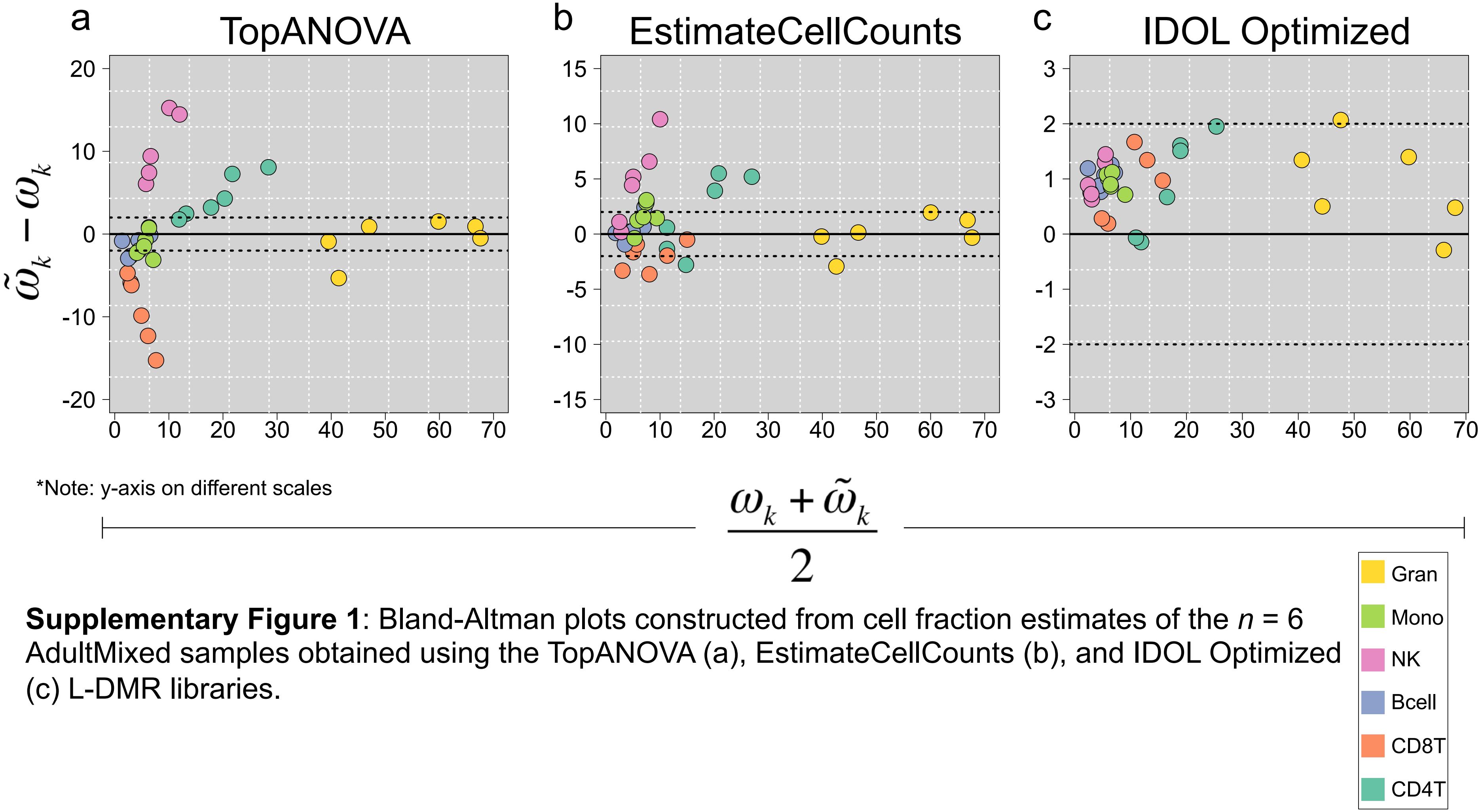

Supplementary Figure 1: Bland-Altman plots constructed from cell fraction estimates of the n = 6 AdultMixed samples obtained using the TopANOVA (a), EstimateCellCounts (b), and IDOL Optimized (c) L-DMR libraries.
