# Supplementary material for: Improving cell mixture deconvolution by identifying optimal DNA methylation libraries (IDOL)
Source: BMC Bioinformatics. 2016 Mar 8;17:120. doi: 10.1186/s12859-016-0943-7 (PMC4782368; doi:10.1186/s12859-016-0943-7)
Supplement: Additional file 4 — Figure S2. Performance metrics for the optimal libraries identified by applying IDOL to the training set. (a) Average R-squared (computed across cell types) in the training set based on the optimal libraries at each assumed library size. (a) Average RMSE (computed across cell types) in the training set based on the optimal libraries at each assumed library size. (PDF 324 kb) [file 12859_2016_943_MOESM4_ESM.pdf]

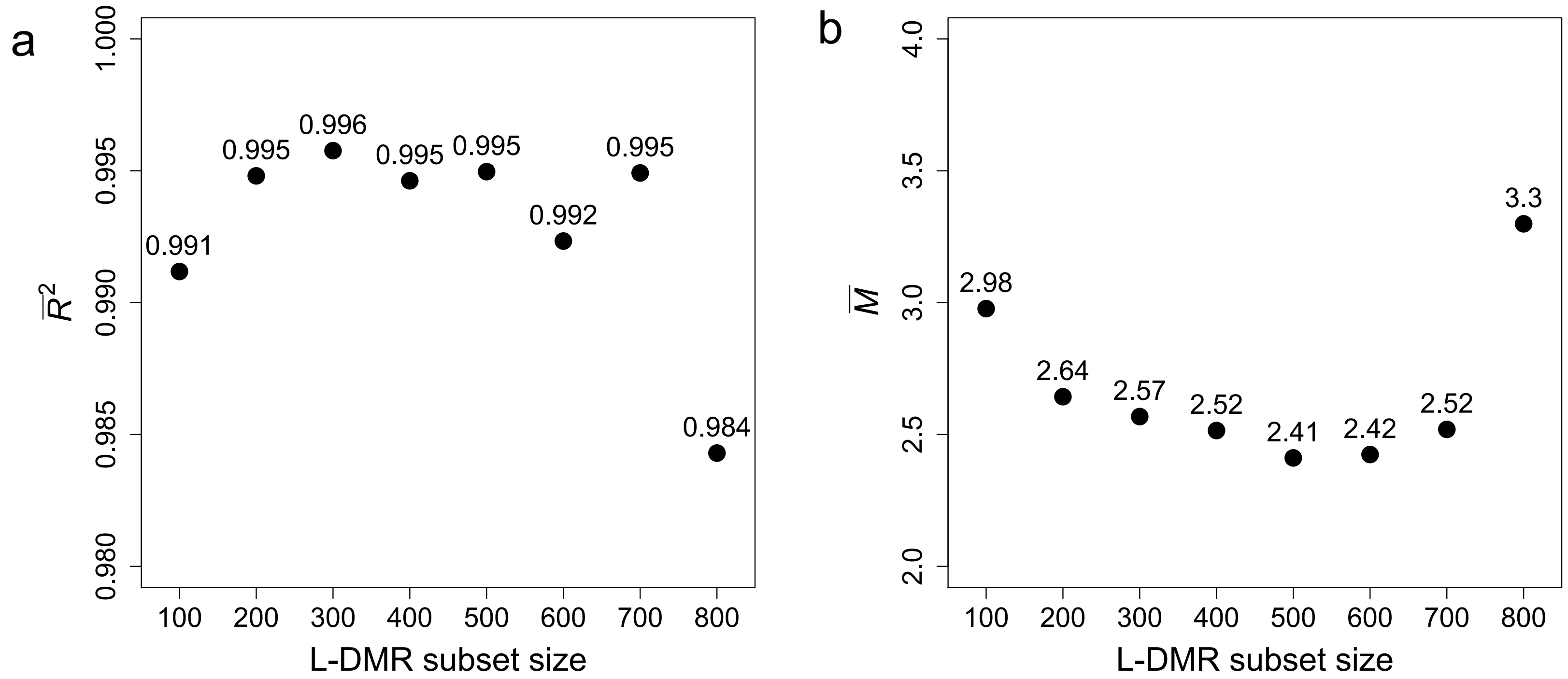

Supplementary Figure 2. Performance metrics for the optimal libraries identified by applying IDOL to the training set. (a) Average R-squared (computed across cell types) in the training set based on the optimal libraries at each assumed library size, i.e., 100, 200, …, 800 CpGs. (a) Average RMSE (computed across cell types) in the training set based on the optimal libraries at each assumed library size, i.e., 100, 200, …, 800 CpGs.
