# Supplementary material for: Improving cell mixture deconvolution by identifying optimal DNA methylation libraries (IDOL)
Source: BMC Bioinformatics. 2016 Mar 8;17:120. doi: 10.1186/s12859-016-0943-7 (PMC4782368; doi:10.1186/s12859-016-0943-7)

Supplementary Figure 5: Cell type prediction performance (R^2 Top and *RMSE* bottom) as a function each cell types mean observed mixture fraction in the MethodA reconstruction samples.

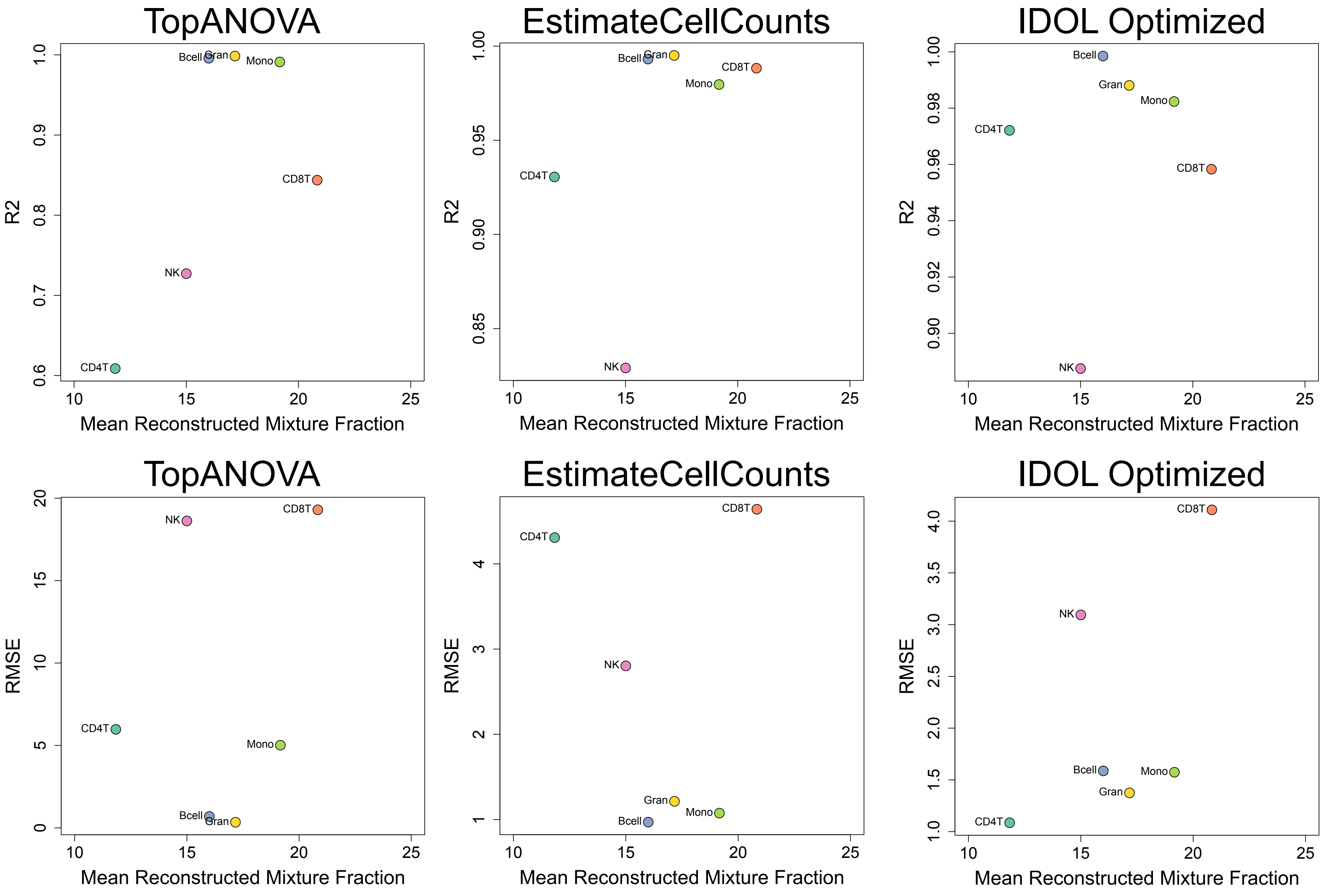

Supplement: Additional file 9 — Figure S5. Cell type prediction performance (R 2 (top) and RMSE (bottom)) as a function of each cell type’s mean observed mixture fraction in the MethodA reconstruction samples. (PDF 698 kb) [file 12859_2016_943_MOESM9_ESM.pdf]
